# Quantifying the combined impacts of anthropogenic CO_2_ emissions and watershed alteration on estuary acidification at biologically-relevant time scales: a case study from Tillamook Bay, OR, USA

**DOI:** 10.3389/fmars.2024.1293955

**Published:** 2024-02-02

**Authors:** Stephen R. Pacella, Cheryl A. Brown, James E. Kaldy, Rochelle G. Labiosa, Burke Hales, T Chris Mochon Collura, George G. Waldbusser

**Affiliations:** 1 Pacific Coastal Ecology Branch, Pacific Ecological Systems Division, Center for Public Health and Environmental Assessment, Office of Research and Development, United States Environmental Protection Agency, Newport, OR, United States,; 2 Region 10, United States Environmental Protection Agency, Seattle, WA, United States,; 3 College of Earth, Ocean, and Atmospheric Sciences, Oregon State University, Corvallis, OR, United States

**Keywords:** ocean acidification, climate change, CO_2_ emissions, water quality, estuary, assessment

## Abstract

The impacts of ocean acidification (OA) on coastal water quality have been subject to intensive research in the past decade, but how emissions-driven OA combines with human modifications of coastal river inputs to affect estuarine acidification dynamics is less well understood. This study presents a methodology for quantifying the synergistic water quality impacts of OA and riverine acidification on biologically-relevant timescales through a case study from a small, temperate estuary influenced by coastal upwelling and watershed development. We characterized the dynamics and drivers of carbonate chemistry in Tillamook Bay, OR (USA), along with its coastal ocean and riverine end-members, through a series of synoptic samplings and continuous water quality monitoring from July 2017 to July 2018. Synoptic river sampling showed acidification and increased ${\text{CO}}_{2}$ content in areas with higher proportions of watershed anthropogenic land use. We propagated the impacts of 1). the observed riverine acidification, and 2). modeled OA changes to incoming coastal ocean waters across the full estuarine salinity spectrum and quantified changes in estuarine carbonate chemistry at a 15-minute temporal resolution. The largest magnitude of acidification (−1.4 ${\text{pH}}_{⊤}$ units) was found in oligo- and mesohaline portions of the estuary due to the poor buffering characteristics of these waters, and was primarily driven by acidified riverine inputs. Despite this, emissions-driven OA is responsible for over 94% of anthropogenic carbon loading to Tillamook Bay and the dominant driver of acidification across most of the estuary due to its large tidal prism and relatively small river discharges. This dominance of ocean-sourced anthropogenic carbon challenges the efficacy of local management actions to ameliorate estuarine acidification impacts. Despite the relatively large acidification effects experienced in Tillamook Bay (−0.16 to −0.23 $\text{p}{\text{H}}_{⊤}$ units) as compared with typical open ocean change (approximately −0.1 ${\text{pH}}_{⊤}$ units), observations of estuarine ${\text{pH}}_{⊤}$ would meet existing state standards for ${\text{pH}}_{⊤}$. Our analytical framework addresses pressing needs for water quality assessment and coastal resilience strategies to differentiate the impacts of anthropogenic acidification from natural variability in dynamic estuarine systems.

## 1 Introduction

Coastal acidification is broadly defined as the lowering of pH and carbonate mineral saturation states in coastal waters resulting from human activities, including fossil fuel combustion, land use change, and eutrophication (Kelly et al., 2011; Cai et al., 2021). The combined effects of these activities in the coastal zone can amplify acidification (Cai et al., 2011; Waldbusser et al., 2011; Cai et al., 2021), while reduced buffering capacities of estuarine waters (Egleston et al., 2010) result in acidification rates which can outpace those of the open ocean (Provoost et al., 2010; Carstensen et al., 2018; Cai et al., 2021). Coastal acidification can harm coastal organisms through physiological and behavioral mechanisms (e.g. Gazeau et al., 2013; Busch and McElhany, 2016; Bednarsek et al., 2019) and result in negative impacts to aquaculture (Barton et al., 2015) and alter the structure and function of coastal food webs (Ainsworth et al., 2011; Busch et al., 2013; Marshall et al., 2017). The many acidification mechanisms in estuarine systems provide both challenges and opportunities for local management options (Kelly et al., 2011). Developing effective management strategies and evaluating decision tradeoffs to address coastal acidification will require the ability to attribute and quantify the mechanisms controlling acidification rates (Strong et al., 2014). Differentiating the anthropogenic impacts of acidification from naturally acidic or corrosive conditions can be challenging in biophysically dynamic coastal systems, but methodologies capable of this are necessary for water quality managers to conduct assessments and establish background conditions (e.g., State of Oregon Department of Environmental Quality, 2023).

There remains a need to better characterize how anthropogenic alterations to the chemistry of freshwaters delivered to the coastal zone interact with acidified ocean waters (Li et al., 2023), which has been recently highlighted in studies of freshwater salinization syndrome in the United States (Kaushal et al., 2017). Watershed delivery of alkalinity and inorganic carbon is known to modulate coastal ${\text{pH}}_{\text{T}}$ and ${\text{CaCO}}_{3}$ saturation states (Kaushal et al., 2017; Van Dam and Wang, 2019; Cai et al., 2021), but there have been limited studies which quantify the magnitude and timing of these watershed effects on coastal acidification and water quality degradation (Raymond and Hamilton, 2018). Anthropogenic changes to watersheds and the resultant changes to riverine chemistry can lead to eutrophication-enhanced coastal acidification (Cai et al., 2011; Hu et al., 2015; Laurent et al., 2018), but can also drive long-term coastal basification due to enhanced riverine alkalinity delivery (Kaushal et al., 2017) and primary production (Borges and Gypens, 2010; Duarte et al., 2013). To date, there is no agreed-upon methodology for predicting how urbanization, agricultural development, climate change, and other human activities alter riverine chemistry and interact with acidified ocean waters in coastal and estuarine habitats.

This study used synoptic sampling and continuous water quality monitoring data to characterize the estuarine water quality impacts of both anthropogenic ${\text{CO}}_{2}$ emissions-driven ocean acidification (OA) and anthropogenic impacts to riverine waters delivered to Tillamook Bay, Oregon (USA), a small open-coast estuary in the northern California Current Large Marine Ecosystem. Acidification research in California Current estuaries has focused on the dominance of high ${\text{CO}}_{2}$ upwelled water in these systems, and limited information is available on how human land use change in coastal watersheds have altered the typically poorly-buffered carbonate systems of small mountainous rivers discharging to these estuaries. Tillamook Bay is subject to coastal upwelling, receives riverine discharges from watersheds which have been modified by agriculture and silviculture (Shanks et al., 2006), provides critical habitat for commercial shellfish aquaculture operations (Tillamook Estuaries Partnership, 2015) and is considered an estuary of national significance as part of the US Environmental Protection Agency’s National Estuary Program. The estuary’s history of bacterial contamination and hypoxia issues resulting from watershed activities (Sullivan et al., 2005) and exposure to naturally high ${\text{CO}}_{2}$, low pH ocean waters due to coastal upwelling, made the estuary an ideal system to investigate both ocean- and land-based drivers of acidification.

## 2 Methods

### 2.1 Study site

**FIGURE 1:**
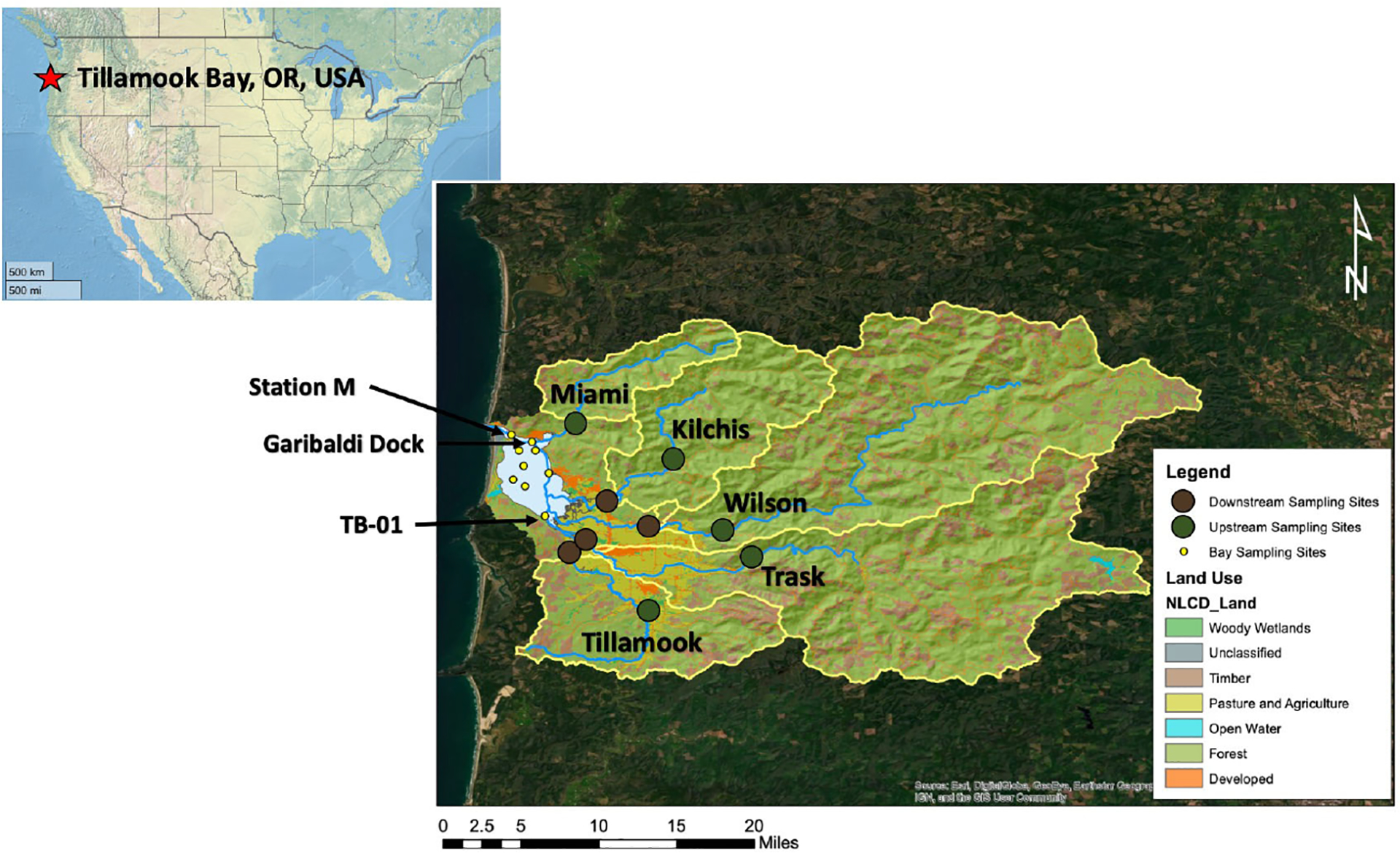
Map of the Tillamook Bay, OR study area. The Tillamook Bay watershed is highlighted, with each river’s sub-watersheds denoted by the yellow boundaries. Watershed land use types are from the National Land Cover Database and color-coded as indicated in the legend. Synoptic sampling stations are indicated by yellow (bay sites), green (upriver sites), and brown (downriver sites) circles. The five major rivers entering the estuary are indicated by blue lines. Garibaldi Dock and TB-01 water quality monitoring stations, as well as the inlet station “M”, are indicated by arrows

Tillamook Bay is a shallow (~2 m average depth), relatively small ($38{\;\text{km}}^{2}$) drowned river mouth estuary on the coast of Oregon, USA with a watershed:estuary ratio of 36.6 (Bricker et al., 2007). Strong tidal forcing [mean tidal volume is $1.23\times 10^{8}\;{\text{m}}^{3}\;{\text{d}}^{-1}$; (Shirzad et al., 1988)], shallow depth, and 1.7 m tidal range result in flushing times on the order of one day to a few weeks, depending on tides and season (Colbert and McManus, 2003; Bricker et al., 2007). The coastal ocean end-member is subject to seasonal upwelling typical of the California Current system, with summers characterized by delivery of cold, salty, high ${\text{CO}}_{2}$, high nutrient, low pH, low dissolved oxygen oceanic waters (Feely et al., 2016), and winters characterized by downwelling and flashy river inputs (Wheatcroft et al., 2013). There are five rivers draining into the estuary: the Miami, Kilchis, Wilson, Trask, and Tillamook rivers in order from north to south (Figure 1). Their combined annual average discharge is $\sim 7.6\times 10^{6}\;{\text{m}}^{3}\;{\text{d}}^{-1}$, with a greater than 2 orders of magnitude difference between wet winter and dry summer months. The watersheds of all five rivers lie in the Oregon Coast Range and drain similar geologic features consisting of Tertiary marine sediments and volcanic rocks (Komar et al., 2004). Land use in the watersheds is subject to human activities via managed forest lands and commercial timber harvest in the upper watersheds, and agricultural activity primarily in support of dairy farming in the lower watersheds. Land cover within the Tillamook Bay watershed is classified as 2% open water, 6% developed, 70% forest, 15% shrub/scrub and grassland/herbaceous, 3% pasture/hay, <1% cultivated crops, and 3% wetlands (https://www.mrlc.gov/data/nlcd-2016-land-cover-conus; Table 1). Most of the agricultural and developed areas are within the lower watersheds of the Wilson, Kilchis, Trask, and Tillamook Rivers, while the Miami River watershed contains relatively little agricultural land cover and is primarily forested.

Tillamook Bay is Oregon’s largest commercial Pacific Oyster (*Crassostrea gigas*) producer by area and value, and provides juvenile habitat for salmon and steelhead (Tillamook Estuaries Partnership, 2015). The upper estuary has a history of bacterial contamination and hypoxia issues resulting from a combination of wastewater treatment plant discharge, agricultural runoff, septic system discharge, and direct animal inputs (Sullivan et al., 2005).

### 2.2 Synoptic water quality surveys

Ten synoptic water quality surveys were conducted in Tillamook Bay and its watershed approximately monthly from July 2017 through July 2018 across eight estuary stations, one coastal ocean/inlet site, and 9 riverine sites both above and below areas with significant agricultural land uses (Figure 1). The inlet station (“M”) was sampled to collect incoming coastal ocean waters on flood tides and was used as the coastal ocean end-member. The upriver sampling stations were located in forested sections of the watersheds and selected to minimize urban and agricultural influence upriver of these sites. Downriver sampling stations were in areas of urban and agricultural land use and as far downstream as possible to sample all stations in a single day without tidal salt intrusion. The Miami River was sampled at only one downriver station as the watershed is primarily forested and previous pilot work had shown minimal downriver gradients in water chemistry. For all seasonal analyses, the dry season included surveys conducted from May – September, and the wet season included surveys from October – April.

Water samples at all stations were collected with Niskin (estuary stations) or Van-Dorn (river stations) gas-tight water sampling devices. Water was sampled from ~1 m depth at all estuary stations except for the inlet station, which was sampled at ~5-m depth and always below any observed surface stratification. A YSI 6000 series sonde was used to measure *in-situ* salinity, temperature, ${\text{pH}}_{\text{NBS}}$ (at *in-situ* temperature), dissolved oxygen, and depth of the sample waters at each station. Sample water for ${\text{CO}}_{2}$ analysis was transferred to triplicate 330-mL amber glass bottles using bestpractices for dissolved gas sampling (e.g. Riebesell et al., 2010), immediately poisoned with 30-μL of a saturated mercuric chloride solution, and sealed with urethane-lined crimp sealed metal caps (Hales et al., 2017). Water for nitrate $\left({{\text{NO}}_{3}}^{-}\right)$ analysis was filtered through a pre-rinsed 0.4μm capsule filter, collected in triplicate polyethylene centrifuge tubes, stored on ice in the field, and frozen within 12 hours of collection. ${{\text{NO}}_{3}}^{-}$ concentrations were colorimetrically determined by flow injection analysis at the Marine Chemistry Laboratory at the University of Washington, WA, USA. Analysis of paired partial pressure of ${\text{CO}}_{2}\;\left(p{\text{CO}}_{2}\right)$ and dissolved inorganic carbon (DIC) for water samples was conducted at Oregon State University in the laboratory of Burke Hales using non-dispersive infrared absorption, as described by Hales et al. (2004) and Bandstra et al. (2006). The full carbonate system [including alkalinity (Alk), ${\text{pH}}_{\text{T}}$ and aragonite saturation state $\left.\left(\Omega_{\text{arag}}\right)\right]$ was calculated utilizing these paired $p{\text{CO}}_{2}$ and DIC measurements, along with *in-situ* salinity and temperature following procedures outlined in Hales et al. (2017). We used CO2SYS [Matlab version 3.1; Lewis and Wallace (1998)], ${\text{K}}_{1}$ and ${\text{K}}_{2}$ constants of Millero (2010), ${\text{KSO}}_{4}$ constants of Dickson et al. (1990), and the borate:salinity relationship of Lee et al. (2010) for all carbonate system calculations.

The ocean end-member specific to each survey was characterized by sampling incoming flood tide waters at station M. River DIC and Alk end-members $\left.({\text{DIC}}_{\text{fwa,down}}\&{\text{Alk}}_{\text{fwa},\text{down}}\right)$ were calculated for each survey using observations from the downriver stations, following the flow-weighted average technique used by Colbert and McManus (2003). Mean daily river discharge for each month of the study was downloaded from the Oregon Water Resources Department Near Real Time Hydrographics Data portal (https://apps.wrd.state.or.us/apps/sw/hydro_near_real_time/). River discharge magnitude and seasonal variation during our 2017–2018 study period was similar (within 5%) to longer term 1996–2014 dynamics, making our observations generally representative of freshwater delivery to Tillamook Bay (Supplementary Figure S1). During some surveys, salt was present at the downriver stations (salinity typically<2), which made those samples unrepresentative of the true river end-member chemistry. To estimate these stations’ DIC and Alk values at zero salinity, linear regressions of salinity versus observed DIC and Alk were created with data from the ocean end-member and the downriver station for that survey. Estimated downriver DIC and Alk values for that station were extrapolated as the y-intercept of these regressions at zero salinity and used for all analyses (Supplementary Figure S2). This regression procedure was required for 8 of the 50 total downriver station sampling events.

### 2.3 Quantifying drivers of Tillamook Bay carbonate chemistry

**FIGURE 2:**
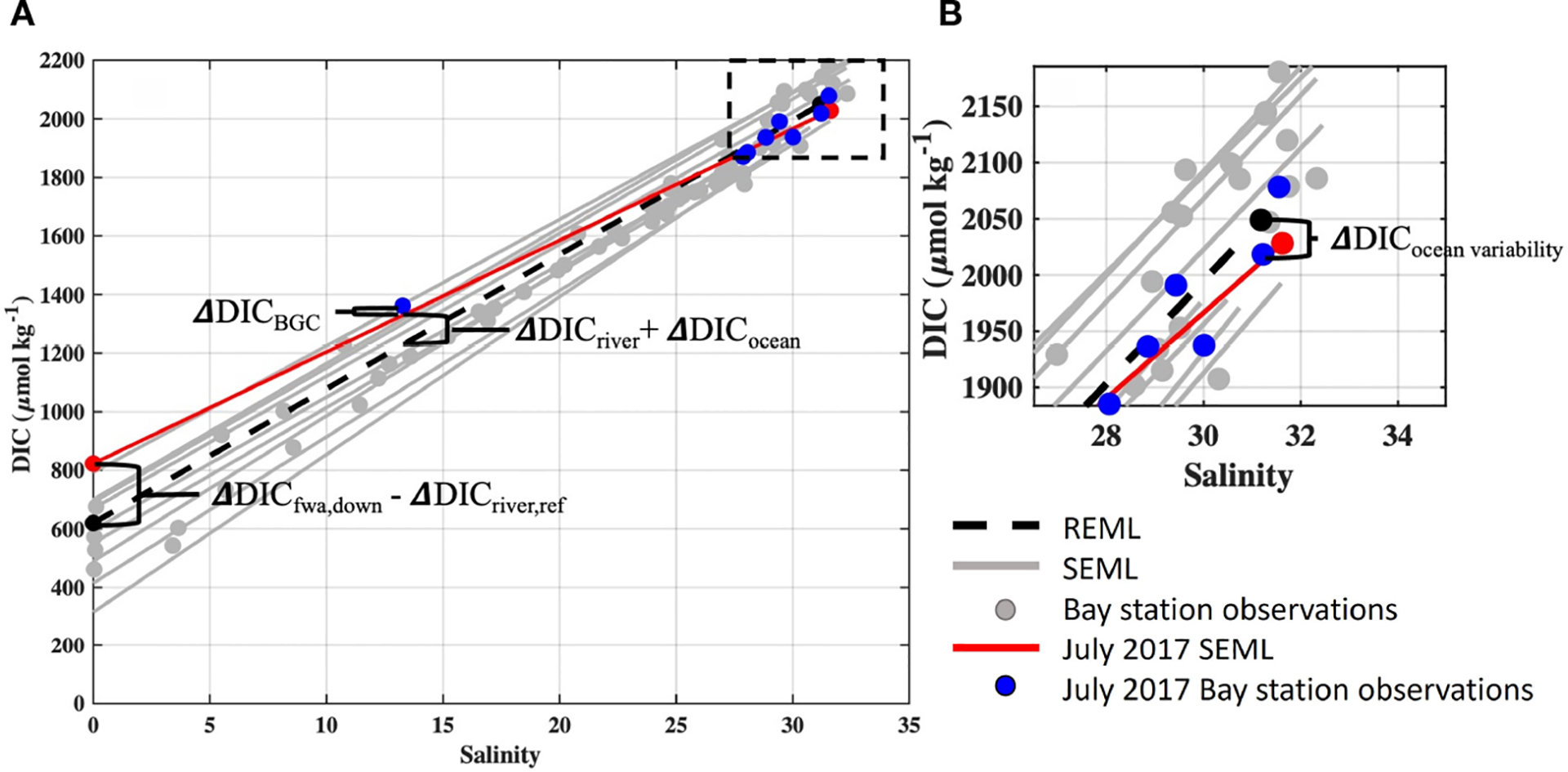
Tillamook Bay estuarine mixing lines for DIC observations. **(A)**. The reference estuarine mixing line (REML) is indicated by black dotted line, synoptic survey-specific estuarine mixing lines (SEML) for all surveys are shown in grey, bay synoptic observations are shown by grey circles, and data for the July 7, 2017 cruise’s SEML (red line) and bay observations (blue circles) are highlighted. Annotations illustrate calculations relevant for mechanistic modeling from Methods Section 2.3. **(B)**. Inset displays ocean end-members of SEMLs and the REML, with the DIC variability attributable to coastal ocean end-member variability ${(\Delta \text{DIC}}_{\text{ocean variability}})$ indicated.

The processes responsible for Tillamook Bay carbonate system variability were calculated using observations from the synoptic water quality surveys (Figure 2). Prior studies of estuarine carbonate system drivers have traditionally quantified departures of pH, $p{\text{CO}}_{2}$, and $\Omega_{\text{arag}}$ from a defined reference station, such as a coastal ocean end-member (e.g. Rheuban et al., 2019). Due to strong tidal forcing and short water residence times in Tillamook Bay we created a reference estuarine mixing line (REML) using average observed ocean and flow-weighted average river end-member DIC (${\text{DIC}}_{\text{ocean,ref}}$), Alk (${\text{Alk}}_{\text{ocean,ref}}$), temperature (${\text{T}}_{\text{ocean,ref}}$), and salinity $\left.({\text{S}}_{\text{ocean,ref}}\right)$ from all synoptic surveys. The REML for DIC and Alk were calculated as:

$$DIC_{\text{REML}}=45.84*S+620$$

$$Alk_{\text{REML}}=53.55*S+508$$

The use of a reference mixing line instead of a single reference station quantifies the role of these mechanisms in altering estuarine chemistry at the observed salinities during the synoptic surveys. This methodology also makes the calculated departures from the reference line directly comparable to estimated changes in estuarine chemistry from OA and riverine carbon enrichments at the same observed salinities.

Departures of pH, $p{\text{CO}}_{2}$, and $\Omega_{\text{arag}}$ from the REML were calculated for each estuary sampling station on each cruise and attributed to four primary processes: coastal ocean end-member variability, river end-member variability, thermal effects, and estuarine biogeochemical cycling. Departures were calculated using the anomalies (e.g. $\Delta {\text{DIC}}_{\text{ocean}}$ and $\Delta {\text{Alk}}_{\text{ocean}}$) between observed and reference values for both DIC and Alk, as described below. These anomalies were added to the observed DIC and Alk at the estuary station, and the full carbonate system was recalculated at observed temperature and salinity. Changes in ${\text{pH}}_{\text{T}},{p\text{CO}}_{2}$, and $\Omega_{\text{arag}}$ due to each process were found as these recalculated values minus the observed values.

#### 2.3.1 Coastal ocean end-member variability

The Oregon coastal ocean is affected by seasonal upwelling dynamics and known for large seasonal differences in carbonate chemistry. Changes to Tillamook Bay carbonate chemistry resulting from coastal ocean end-member variability were estimated for each survey by comparing survey-specific estuarine mixing lines (SEML) to the averaged REML for both DIC and Alk (Figure 2). The variability of coastal ocean end-member DIC and Alk were calculated as the differences between the SEML and REML at the observed salinity of Station M $\left.({\text{S}}_{\text{M}}\right)$ (Figure 2). To account for the small differences between ${\text{S}}_{M}$ and ${\text{S}}_{\text{ocean,ref}}$, we used the REML to calculate DIC and ALK of the REML at ${\text{S}}_{\text{M}}$ and used these values to represent the average ocean end-member. The differences in the survey-specific DIC (${\text{DIC}}_{\text{M}}$) and Alk $\left({\text{Alk}}_{\text{M}}\right)$ at station M from average ocean conditions were calculated as

$$\Delta DIC_{\text{ocean variability}}=DIC_{M}-DIC_{\text{REML},SM}$$

$$\Delta Alk_{\text{ocean variability}}=Alk_{M}-Alk_{\text{REML},SM}$$

where $DIC_{\text{REML},SM}$ and $Alk_{\text{REML},SM}$ were found by solving the reference mixing line equations at ${\text{S}}_{\text{M}}$, and $\Delta D{IC}_{\text{ocean variability}}$ and $\Delta Alk_{\text{ocean variabilty}}$ are the variability of DIC and Alk of the coastal ocean end-member relative to average conditions. The effect of this variability was propagated to the estuary synoptic survey observations while accounting for freshwater dilution:

$$\Delta DIC_{\text{ocean}}=\frac{S_{\text{obs}}}{S_{M}}\times \Delta DIC_{\text{ocean variability}}$$

$$\Delta Alk_{\text{ocean}}=\frac{S_{\text{obs}}}{S_{M}}\times \Delta Alk_{\text{ocean variability}}$$

where $S_{\text{obs}}$ is the observed salinity at each estuary survey station.

#### 2.3.2 River end-member variability

Changes to observed estuarine carbonate chemistry due to river end-member variability were estimated by calculating differences in observed ${\text{DIC}}_{\text{fwa,down}}$ and ${\text{Alk}}_{\text{fwa,down}}$ for each survey from the averaged river reference value (${\text{DIC}}_{\text{river},\text{ref}}$ and ${\text{Alk}}_{\text{river,ref}}$):

$$\Delta DIC_{\text{river}}=\left(1-\frac{S_{obs}}{S_{M}}\right)\times \left(DIC_{fwa,\text{down}}-DIC_{\text{river},\text{ref}}\right)$$

$$\Delta Alk_{\text{river}}=\left(1-\frac{S_{obs}}{S_{M}}\right)\times \left(Alk_{fwa,\text{down}}-Alk_{\text{river},\text{ref}}\right)$$

#### 2.3.3 Thermal effects

The effects of warming and cooling within the estuary were found by recalculating the estuarine carbonate system at each station and for each survey using observed DIC, Alk, S, and the reference mixing line temperature at the observed salinity (i.e. ${\text{T}}_{\text{ref},\text{Sobs}}$). Changes in pH, ${p\text{CO}}_{2}$, and $\Omega_{\text{arag}}$ due to thermal effects were found as these recalculated values minus the observed values.

#### 2.3.4 Estuarine biogeochemical cycling

The impacts of local biogeochemical cycling on estuarine carbonate chemistry were estimated as the departures of DIC and Alk ($\Delta {\text{DIC}}_{\text{BGC}}$ and $\Delta {\text{Alk}}_{\text{BGC}}$, respectively) observed at each station from the cruise-specific conservative mixing values of DIC and Alk at the same salinity as the observations (i.e. ${\text{DIC}}_{\text{cons,S}}$ and ${\text{Alk}}_{\text{cons,S}}$):

$$DIC_{\text{cons},S}=\frac{S_{obs}}{S_{M}}\times DIC_{M}+\left(1-\frac{S_{obs}}{S_{M}}\right)\times \left(DIC_{fwa,\text{down}}\right)$$

$$Alk_{\text{cons},S}=\frac{S_{obs}}{S_{M}}\times Alk_{M}+\left(1-\frac{S_{obs}}{S_{M}}\right)\times \left(Alk_{fwa,\text{down}}\right)$$

$$\Delta DIC_{BGC}=DIC_{\text{obs}}-DIC_{\text{cons},S}$$

$$\Delta Alk_{BGC}=Alk_{\text{obs}}-Alk_{\text{cons},S}$$

#### 2.3.5 Garibaldi Dock and TB-01 continuous water quality monitoring

Two water quality monitoring stations were established in July 2017; the Garibaldi Dock station in the polyhaline portion of the estuary, and the TB-01 station in the mesohaline portion of the estuary (Figure 1). Both stations consisted of a YSI 6000 series sonde sampling at a 15-minute interval for salinity, temperature, pressure, ${\text{pH}}_{\text{NBS}}$ (at *in-situ* temperature), and dissolved oxygen. The Garibaldi Dock station sonde was located ~1.5 m above the benthos and ranged in depth from 1.5 m to 5.9 m depending upon tidal stage, while the TB-01 station sonde was anchored ~0.2 m above the benthos and ranged in depth from 0.2 m to 3.6 m. Deployed sondes were replaced every 2 – 4 weeks with a freshly calibrated sonde, and laboratory tests were performed immediately following deployments to quantify instrument drift and identify possible fouling issues. The pH probes were calibrated using NIST standards of ${\text{pH}}_{\text{NBS}}$ 7 and 10 following manufacturer recommendations. The ${\text{pH}}_{\text{NBS}}$ readings from the sondes were converted to the Total scale $\left({\text{pH}}_{\text{T}}\right)$ using CO2SYS v3.1, and all reported monitoring observations in this manuscript refer to these converted ${\text{pH}}_{\text{T}}$ values. The synoptic estuary water quality surveys sampled directly adjacent to both sondes during the surveys, and calculated ${\text{pH}}_{\text{T}}$ from discrete samples was used as an *in-situ* validation of the sondes’ ${\text{pH}}_{\text{T}}$ readings. Root mean squared error between calculated ${\text{pH}}_{\text{T}}$ from discrete bottle samples (using $p{\text{CO}}_{2}$ and DIC) and converted YSI ${\text{pH}}_{\text{T}}$ was 0.07 (n = 7) at the Garibaldi Dock, and 0.13 (n=7) at TB-01. We note these discrepancies between the sensor data and bottle data include sampling and analytical uncertainties in addition to the uncertainty specific to the accuracy of the sensor itself.

An alkalinity-salinity regression (Supplementary Figure S3) was created using all discrete bottle samples (n = 89) from the estuary surveys with calculated alkalinity from the paired $p{\text{CO}}_{2}$ and DIC data:

$${\text{Alk}}_{\text{sal}}=55.2*\text{Salinity}+452$$

$$R^{2}=0.989,\;\text{Root mean square error}=51\;\mu \text{mol}\;kg^{-1}.$$

This regression was used to calculate alkalinity time series for Garibaldi Dock and TB-01 stations, and the full carbonate system was then calculated using this ${\text{Alk}}_{\text{sal}}$ and observed ${\text{pH}}_{\text{T}}$ (CO2SYS Matlab version 3.1). Propagation of the uncertainties in YSI ${\text{pH}}_{\text{T}}$ (0.07 units), ${\text{Alk}}_{\text{sal}}\left(51\;\mu \text{eq}\;{\text{kg}}^{-1}\right.$), salinity, temperature, pressure, and dissociation constants resulted in calculated pCO2 uncertainties of ~17% and calculated $\Omega_{\text{arag}}$ uncertainties of ~14%.

### 2.4 Anthropogenic impacts to Tillamook Bay carbonate chemistry

#### 2.4.1 Estimating coastal ocean anthropogenic carbon

We estimated the impacts of increasing atmospheric ${\text{CO}}_{2}$ levels on anthropogenic carbon concentrations of the coastal ocean end-member (${\text{C}}_{\text{ant}}$) using a modified version of the $\Delta {\text{TCO}}_{2}$ method (Gruber et al., 1996; Pacella et al., 2018; Evans et al., 2019) found in Hare et al. (2020). This method estimated the ventilation age of ocean source waters to the estuary utilizing the observed apparent oxygen utilization (AOU) and assuming an oxygen utilization rate of $5\;\mu \text{mol}\;{\text{O}}_{2}\;{\text{kg}}^{-1}\;{\text{yr}}^{-1}$ specific to North Pacific waters. The coastal ocean waters entering Tillamook Bay are subject to seasonal upwelling and are characterized by a range of water mass ventilation ages and ${\text{C}}_{\text{ant}}$ concentrations (Feely et al., 2016). The benefit of this approach is that it captures the ${\text{C}}_{\text{ant}}$ dynamics resulting from these variable water mass ventilation histories. Estuarine metabolism will also impact AOU calculations and potentially bias ventilation age estimates. Our method described below attempts to minimize this potential issue by filtering observations to only retain the densest water, with the assumption that these waters are most representative of coastal ocean waters recently advected into the estuary. We note that this technique is an inherently conservative technique for estimating ocean acidification impacts as ventilation age estimates are utilized to reduce modern atmosphere-equilibrated ${\text{C}}_{\text{ant}}$ estimates via equilibration with older, lower atmospheric ${\text{CO}}_{2}$ levels. Biases in AOU and ventilation ages would therefore never result in overestimates of ${\text{C}}_{\text{ant}}$ as compared to modern atmosphere-equilibrated values. The anthropogenic carbon concentrations of the coastal ocean end-member were estimated using observations from the Garibaldi Dock monitoring station, given its close proximity to the estuary inlet and exposure to incoming coastal waters on flood tides, as follows:

1. Water densities were calculated for the full time series at the Garibaldi Dock using temperature and salinity. A moving 3-day window was used to identify the observed maximum water density, which was assumed to be representative of coastal ocean waters entering Tillamook Bay, and time series of the corresponding salinity, temperature, density, pH, ${\text{Alk}}_{\text{sal}}$, and ${\text{O}}_{2}$ were created.
2. AOU was calculated at each time point for this ocean end-member time series $\left({\text{AOU}}_{\text{ocean}}\right)$ as:
$$AOU_{\text{ocean}}=O_{2,\text{sat}}-O_{2,\text{maxdens}}$$
  where ${\text{O}}_{2,\text{sat}}$ is the oxygen concentration of the waters in equilibrium with the atmosphere at observed salinities and temperatures in $\mu \text{mol}\;{\text{O}}_{2}\;{\text{kg}}^{-1}$, and ${\text{O}}_{2\text{,maxdens}}$ is the observed ${\text{O}}_{2}$ concentration at the Garibaldi Dock in $\mu \text{mol}\;{\text{O}}_{2}\;{\text{kg}}^{-1}$ corresponding to the observed 3-day moving density maximum.
3. The ventilation ages of these waters $\left({\text{Age}}_{\text{vent}}\right)$ in years were then calculated using the ratio of AOU and oxygen utilization rate as:
$$Age_{\text{vent}}=AOU_{\text{ocean}}/5\;\mu mol\;O_{2}\;{kg}^{-1}\;{yr}^{-1}$$
  ${\text{Age}}_{\text{vent}}$ was set to zero when AOU was negative (i.e. ${\text{O}}_{2}$ was supersaturated with respect to the atmosphere).
4. The year of atmospheric equilibration for the ocean end-member was calculated as:
$${\text{Year}}_{\text{ocean,equil}}=2018-{\text{Age}}_{\text{vent}}$$
  ${\text{Year}}_{\text{ocean,equil}}$ was used to determine the global atmospheric ${\text{CO}}_{2}$ level for that year which surface ocean waters would have equilibrated with (${\text{CO}}_{2,\text{equil,year}}$; Meinshausen et al., 2011). ${\text{CO}}_{2\text{,equil,year}}$ was paired with the ocean end-member ${\text{Alk}}_{\text{sal}}$ to calculate the equilibrated DIC value at the time of last ventilation $\left({\text{DIC}}_{\text{equil,vent}}\right)$ as:
$$DIC_{\text{equil,vent}}\sim f\left({CO}_{2,\text{equil,year}},Alk_{\text{sal}}\right)$$
5. ${\text{C}}_{\text{ant}}$ was calculated using the $\Delta {\text{TCO}}_{2}$ method following Pacella et al., 2018, which maintains the disequilibrium between atmospheric-equilibrated DIC $\left(\text{DI}{\text{C}}_{\text{equil,vent}}\right)$ and *in-situ* seawater DIC values. The DIC disequilibrium value was calculated as:
$$\Delta DIC_{\text{diseq}}=DIC_{\text{obs}}-DIC_{\text{equil,vent}}$$
  where ${\text{DIC}}_{\text{obs}}$ is the 2017/2018 DIC value of the ocean end-member calculated with the pH and ${\text{Alk}}_{\text{sal}}$ time series. The preindustrial DIC value of coastal ocean waters was calculated as:
$$DIC_{\text{ocean},PI}=DIC_{\text{equil},1765}+\Delta DIC_{\text{diseq}}$$
  where ${\text{DIC}}_{\text{equil,}1765}$ is the DIC value of the ocean end-member equilibrated with a year 1765 atmospheric ${\text{CO}}_{2}$ value (278 ppm) The coastal ocean end-member anthropogenic carbon concentration (${\text{C}}_{\text{ant}};\mu \text{mol}\;{\text{kg}}^{-1}$) was calculated as:
$$C_{ant}=DIC_{obs}-DIC_{\text{ocean},PI}$$
  This method assumes no changes in temperature, salinity, and alkalinity.

The Garibaldi Dock monitoring did not start until August 2017, and therefore estimated ocean ${\text{C}}_{\text{ant}}$ values using this procedure were not available for the two July 2017 synoptic surveys. To estimate ocean ${\text{C}}_{\text{ant}}$ for these July 2017 surveys, the same methodology was applied using observations of water density, AOU, salinity, temperature, DIC, and Alk from Station “M” for each survey.

#### 2.4.2 Anthropogenic alterations of river chemistry

Changes to river delivery of DIC and Alk ($\text{DI}{\text{C}}_{\text{enrich}}$ and ${\text{Alk}}_{\text{enrich}}$ respectively) due to anthropogenic development in the lower watershed were assumed to be constrained by the difference between the flow-weighted average concentrations of upriver and downriver station DIC and Alk concentrations for each synoptic survey, such that:

$$DIC_{\text{enrich}}=DIC_{\text{fwa,down}}-DIC_{fwa,up}$$

$$Alk_{\text{enrich}}=Alk_{\text{fwa,down}}-Alk_{\text{fwa,up}}$$

where ${\text{DIC}}_{\text{fwa},\text{up}}$ and, ${\text{DIC}}_{\text{fwa,down}}$ are the flow-weighted average DIC concentrations of the upriver and downriver stations, respectively (in $\mu {\text{mol kg}}^{-1}$), ${\text{Alk}}_{\text{fwa},\text{up}}$ and ${\text{Alk}}_{\text{fwa,down}}$ are the flow-weighted average Alk concentrations of the upriver and downriver stations, respectively (in $\mu {\text{mol kg}}^{-1}$), and ${\text{DIC}}_{\text{enrich}}$ and ${\text{Alk}}_{\text{enrich}}$ are the magnitudes of river end-member DIC and Alk changes due to human activities in the lower watershed, respectively (in $\mu \text{mol}\;{\text{kg}}^{-1}$). While we were able to apply survey-specific observations of watershed ${\text{DIC}}_{\text{enrich}}$ and ${\text{Alk}}_{\text{enrich}}$ to each set of estuary survey observations, the variability of these values amongst surveys prevented us from choosing a single representative value for each to apply to the water quality monitoring time series at the Garibald Dock and TB-01 monitoring stations. We therefore developed a regression between flow-weighted averages of ${\text{DIC}}_{\text{enrich}}$ and ${\text{Alk}}_{\text{enrich}}$ with daily Trask River discharge (the only river with daily gauging for the entire study period) to calculate a time series of daily ${\text{DIC}}_{\text{enrich}}$ and ${\text{Alk}}_{\text{enrich}}$ for the full monitoring period (Supplementary Figure S4). The relationship of Trask River discharge with ${\text{DIC}}_{\text{enrich}}$ and ${\text{Alk}}_{\text{enrich}}$ was calculated as:

$$DIC_{\text{enrich}}=1,576*Q^{-0.4765}$$

$$R^{2}=0.86,\text{root mean square error}=25\;\mu mol\;{kg}^{-1},$$

$$Alk_{\text{enrich}}=1,178*Q^{-0.5458}$$

$$R^{2}=0.86,\text{root mean square error}=15\;\mu mol\;{kg}^{-1},$$

where ${\text{DIC}}_{\text{enrich}}$ and ${\text{Alk}}_{\text{enrich}}$ are daily flow-weighted average downriver additions of DIC and Alk as previously defined with units of $\mu \text{mol}\;{\text{kg}}^{-1}$, and Q is the average daily discharge of the Trask River in cubic feet per second.

To test the sensitivity of our results to this method, we also created individual river regressions of ${\text{DIC}}_{\text{enrich}}$ and ${\text{Alk}}_{\text{enrich}}$ versus discharge for the Wilson, Kilchis, Trask, and Tillamook Rivers utilizing only data with salinity <0.2. This therefore avoided using the zero salinity extrapolations for some of the downriver stations as discussed in Section 2.2. We report these regressions in the Supplementary Material (Supplementary Figure S5) and the estimated impacts to Tillamook Bay acidification dynamics using these regressions in Supplementary Figures S6, S7. Estimated acidification impacts in Tillamook Bay were not significantly altered utilizing this alternate method as compared with using the zero-salinity extrapolations, and we do not discuss the method further in this manuscript.

#### 2.4.3 Calculating impacts to estuary chemistry from ocean and river acidification

Present-day impacts of OA and riverine carbon enrichments to estuarine ${\text{pH}}_{\text{T}},p{\text{CO}}_{2}$, and $\Omega_{\text{arag}}$ were estimated for observations from the synoptic surveys and both water quality monitoring stations using the estimates of ocean ${\text{C}}_{\text{ant}}$, river ${\text{DIC}}_{\text{enrich}}$, and river ${\text{Alk}}_{\text{enrich.}}$. OA impacts were estimated by subtracting ${\text{C}}_{\text{ant}}$ concentrations (corrected for salinity) from the present-day survey and monitoring stations’ DIC observations and recalculating the full carbonate system using this estimate of preindustrial DIC and observed Alk (note that this method assumes no change in alkalinity associated with OA). These recalculated ${\text{pH}}_{\text{T}},\Omega_{\text{arag,}}$ and $p{\text{CO}}_{2}$ values (e.g. ${\text{pH}}_{\text{No OA}}$) were subtracted from present-day observations (e.g. ${\text{pH}}_{\text{obs}}$) to calculate OA-specific impacts to estuarine water quality (e.g. ${\Delta \text{pH}}_{\text{OA}}$):

$$\Delta pH_{OA}=pH_{\text{obs}}-pH_{\text{No OA}}$$

$$\Delta pCO_{2,OA}=p{CO}_{2,obs}-p{CO}_{2,\text{No OA}}$$

$$\Delta \Omega_{\text{arag,OA}}=\Omega_{\text{arag,obs}}-\Omega_{\text{arag},\text{No OA}}$$

Impacts to present-day estuarine chemistry from riverine carbon enrichments were estimated similarly by subtracting ${\text{DIC}}_{\text{enrich}}$ and ${\text{Alk}}_{\text{enrich}}$ from the present-day survey and monitoring stations’ DIC and Alk and recalculating the full carbonate system using these estimates of DIC and Alk absent anthropogenic development of the lower watershed:

$$\Delta pH_{\text{enrich}}=pH_{obs}-pH_{No\;\text{enrich}}$$

$$\Delta pCO_{2,\text{enrich}}=pCO_{2,obs}-pCO_{2,No\;\text{enrich}}$$

$$\Delta \Omega_{\text{arag,enrich}}=\Omega_{\text{arag,obs}}-\Omega_{\text{arag},No\;\text{enrich}}$$

The sum of the impacts to estuarine chemistry from both OA and riverine carbon enrichments were calculated as:

$$DIC_{\text{PI}}=DIC_{obs}-\left[\left(1-\frac{S_{obs}}{S_{M}}\right)*DIC_{\text{enrich}}\right]-\frac{S_{obs}}{S_{M}}*C_{ant}$$

$$Alk_{\text{PI}}=Alk_{obs}-\left[\left(1-\frac{S_{obs}}{S_{M}}\right)*Alk_{\text{enrich}}\right]$$

Where $\text{DI}{\text{C}}_{\text{PI}}$ and $\text{Al}{\text{k}}_{\text{PI}}$ are the estimated DIC and Alk concentrations in the absence of both OA and riverine carbon enrichment. $\text{DI}{\text{C}}_{\text{PI}}$ and $\text{Al}{\text{k}}_{\text{PI}}$ were combined with observed temperature and salinity to recalculate the full carbonate system and estimate present-day anthropogenic impacts to estuarine chemistry as:

$$\Delta DIC_{\text{total}}=DIC_{obs}-DIC_{PI}$$

$$\Delta Alk_{\text{total}}=Alk_{obs}-Alk_{PI}$$

$$\Delta pH_{\text{total}}=pH_{obs}-pH_{PI}$$

$$\Delta pCO_{2,\text{total}}=pCO_{2,obs}-pCO_{2,PI}$$

$$\Delta \Omega_{\text{arag,total}}=\Omega_{\text{arag,obs}}-\Omega_{\text{arag,PI}}$$

Anthropogenic loading of DIC and Alk to Tillamook Bay from ${\text{DIC}}_{\text{enrich}}\left(DIC_{\text{enrich,load}}\right),{\text{ALK}}_{\text{enrich}}\left(Alk_{\text{enrich,load}}\right)$, and ${\text{C}}_{\text{ant}}\left(C_{\text{ant,load}}\right)$ were calculated as:

$$DIC_{\text{enrich,load}}=DIC_{\text{enrich}}*Q_{\text{Trask}}*\frac{Q_{\text{Total}}}{Q_{\text{Trask}}}$$

$$Alk_{\text{enrich,load}}=Alk_{\text{enrich}}*Q_{\text{Trask}}*\frac{Q_{\text{Total}}}{Q_{\text{Trask}}}$$

$$C_{\text{ant,load}}=C_{\text{ant}}*1.23*10^{8}\frac{m^{3}}{d}$$

where $Q_{\text{Trask}}$ is the discharge of the Trask River, $\frac{Q_{\text{Total}}}{Q_{\text{Trask}}}$ is the ratio of total river discharge to Tillamook Bay to Trask River discharge, and $1.23⋆10^{8}\frac{m^{3}}{d}$ is the average tidal volume for Tillamook Bay.

### 2.5 Estimates of watershed nutrient sources and land use

ArcMap 10x Spatial Analyst Hydrology Tools was used to define the sub-watersheds for each of the nine river sampling sites utilizing a flow direction raster and a “pourpoint” to generate a raster of the area that is hydrologically upstream (Figure 1). Land cover within each of these nine sub-watersheds was obtained from the 2016 Multi-Resolution Land Characteristics (MRLC) land cover layer for the conterminous United States. MRLC categories included in the anthropogenic land use grouping for this study were cropland, developed space, pasture/hay, and wetlands. Satellite imagery was used to confirm that lower watershed areas categorized as wetlands were used as dairy pastures. The number of permitted cows within each sub-watershed was estimated from the combined animal feeding operation (CAFO) data provided by the Oregon Department of Agriculture. Sources of nitrogen loading specific to each of the nine river sampling sites was estimated using the SPAtially Referenced Regressions On Watershed attributes (SPARROW) model developed by the United States Geological Survey (Wise and Johnson, 2011). The SPARROW model is a hybrid statistical and mechanistic model which uses spatial data describing watershed attributes to predict water quality of a surface water network and estimate the discharges of nutrients to streams. For this study, nitrogen loads from forest lands, livestock manure (included confined cattle and grazing livestock), point sources associated with wastewater facilities, non-sewered areas, runoff from developed lands, farm fertilizer, atmospheric deposition, and red alder were estimated to each river sampling site.

## 3 Results

### 3.1 Seasonal carbonate chemistry dynamics in Tillamook Bay and end-members

#### 3.1.1 River observations

**FIGURE 3:**
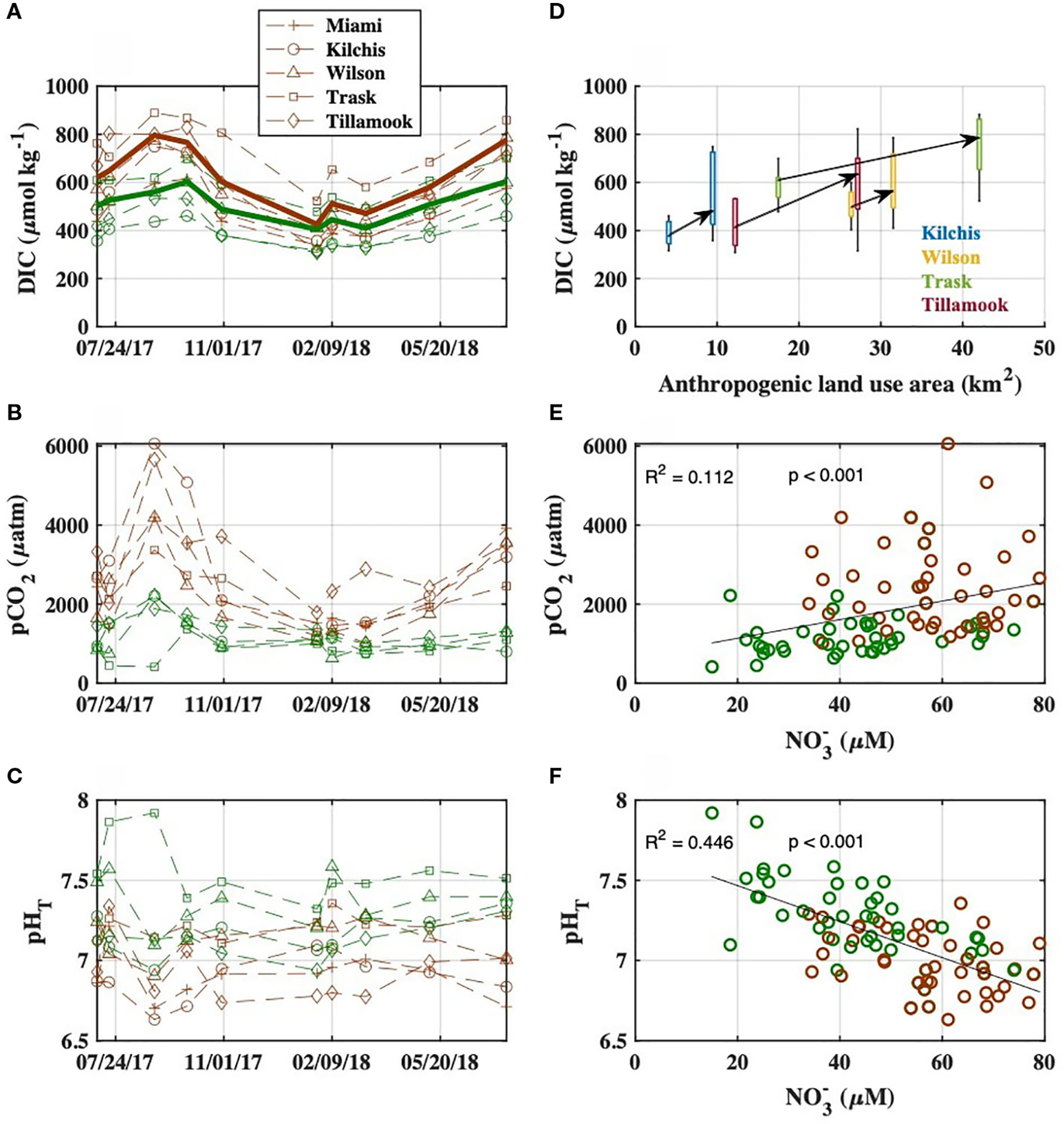
**(A-C)** Observed carbonate system variables at upstream (green) and downstream (brown) river stations during the synoptic surveys from July 2017 to July 2018. Bold lines indicate flow-weighted averages in **(A)**. **(D)** The relationships between anthropogenic land use area (from MRLC data) in the sub-watershed of each sampling site and river DIC concentrations. Data are color coded by river, and arrows connect upriver to downriver median DIC concentrations for each river Bivariate plots and linear regressions of riverine **(E)** ${p\text{CO}}_{2}$ (linear regression performed between ${{\text{NO}}_{3}}^{-}$ and log-transformed ${p\text{CO}}_{2}$ to correct for heteroscedasticity) and **(F)** $\text{p}{\text{H}}_{\text{T}}$ with ${{\text{NO}}_{3}}^{-}$ concentrations, with upstream stations in blue and downstream stations in red. Regression between ${{\text{NO}}_{3}}^{-}$ and log-transformed $p{\text{CO}}_{2}$ is displayed in ${p\text{CO}}_{2}$ space for clarity.

In all four rivers with upstream and downstream sampling stations, we observed downstream increases in DIC, Alk, the ratio of DIC : Alk, $p{\text{CO}}_{2}$, and ${{\text{NO}}_{3}}^{-}$, and decreases in ${\text{pH}}_{\text{T}}$ (Figures 3A–C; Supplementary Figure S8). These downstream changes in river chemistry were most pronounced during the dry season and occurred in areas with increased human land use modifications (Figure 3D, Supplementary Figure S8) as well as higher proportions of anthropogenic nitrogen sources as determined by SPARROW (Figure S8). The rivers displayed different carbonate chemistry conditions at the upstream stations before entering the lower watershed (Figures 3A–C), but patterns of upstream to downstream carbonate system changes were consistent across rivers (Figure 3D, Supplementary Figure S8). River ${\text{pH}}_{\text{T}},{p\text{CO}}_{2}$, and DIC : Alk ratios were significantly correlated with river ${{\text{NO}}_{3}}^{-}$ levels (Figures 3E, F, Supplementary Figure S8; Supplementary Table S1). DIC and Alk concentrations at all river stations were highest during the dry season and lowest during the wet season (Figure 3A, Supplementary Table S1). Upriver DIC ranged from 309 to $700\;\mu \text{mol}\;{\text{kg}}^{-1}$, and Alk from 231 to $653\;\mu \text{mol}\;{\text{kg}}^{-1}$, with the Trask River having the highest average concentrations. Downriver DIC ranged from 315 to $883\;\mu \text{mol}\;{\text{kg}}^{-1}$, and Alk from 212 to $756\;\mu \text{mol}\;{\text{kg}}^{-1}$, with the Trask River again having the highest average concentrations.

**FIGURE 4:**
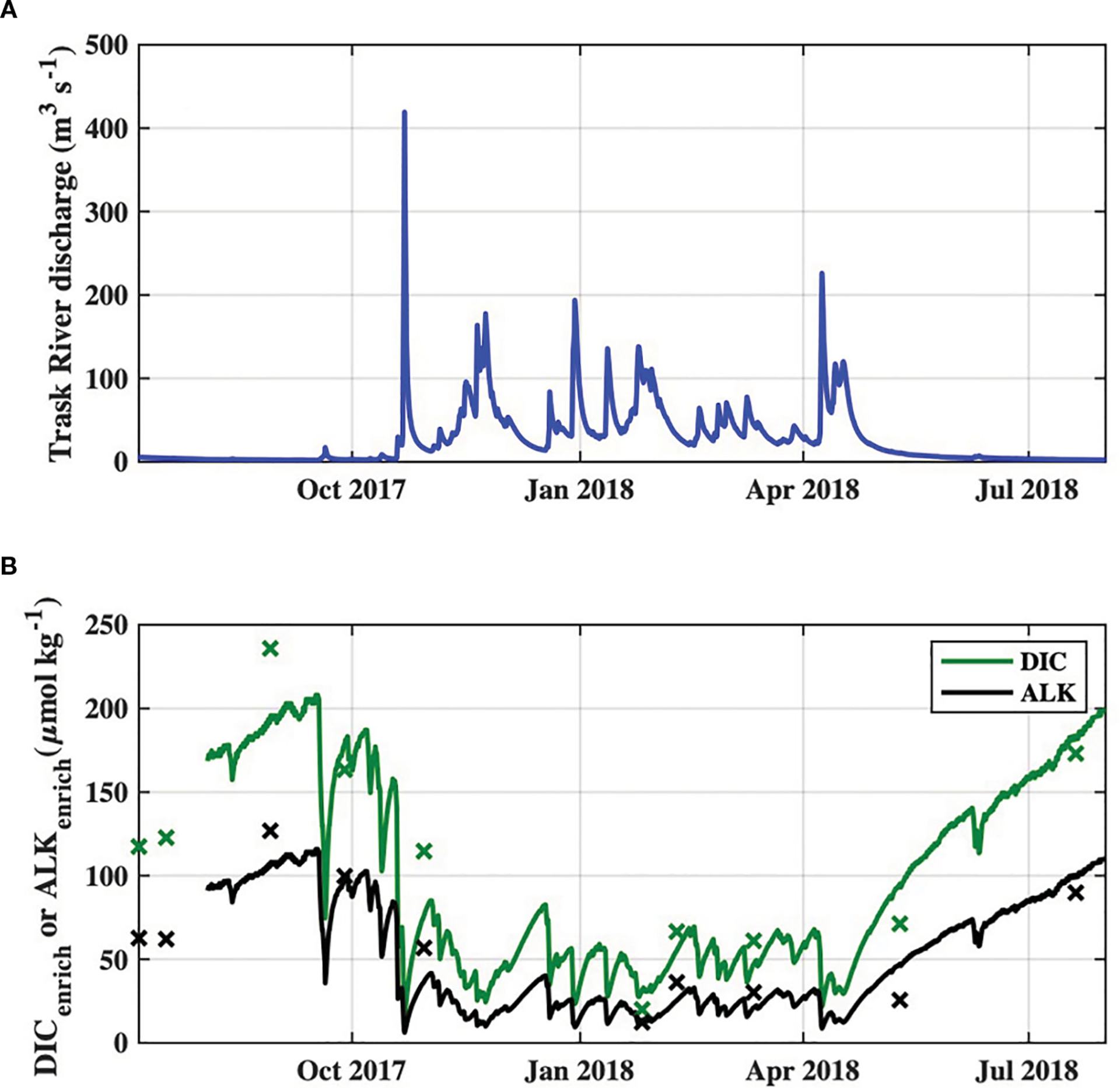
Time series of **(A)** observed Trask River discharge from USGS station 14302480, and **(B)** DIC_enrich_ and Alk_enrich_ of river end-member discharges to the Tillamook Estuary during the study period. Survey-specific observations of DIC_enrich_ and Alk_enrich_ are indicated by “x” markers.

Increases in downstream flow-weighted averages of river DIC and Alk, i.e. ${\text{DIC}}_{\text{enrich}}$ and ${\text{Alk}}_{\text{enrich}}$, were significantly correlated with Trask River discharge on the day of sampling (Supplementary Figure S4) and were highest during periods of low river discharge (Figure 4). ${\text{DIC}}_{\text{enrich}}$ concentrations were often much larger than ocean ${\text{C}}_{\text{ant}}$ values during the study period, but were largely composed of ${{\text{HCO}}_{3}}^{-}$ and therefore contributed to increased ${\text{Alk}}_{\text{enrich}}$ values as well.

#### 3.1.2 Coastal ocean observations and estimated ocean acidification signals

**FIGURE 5:**
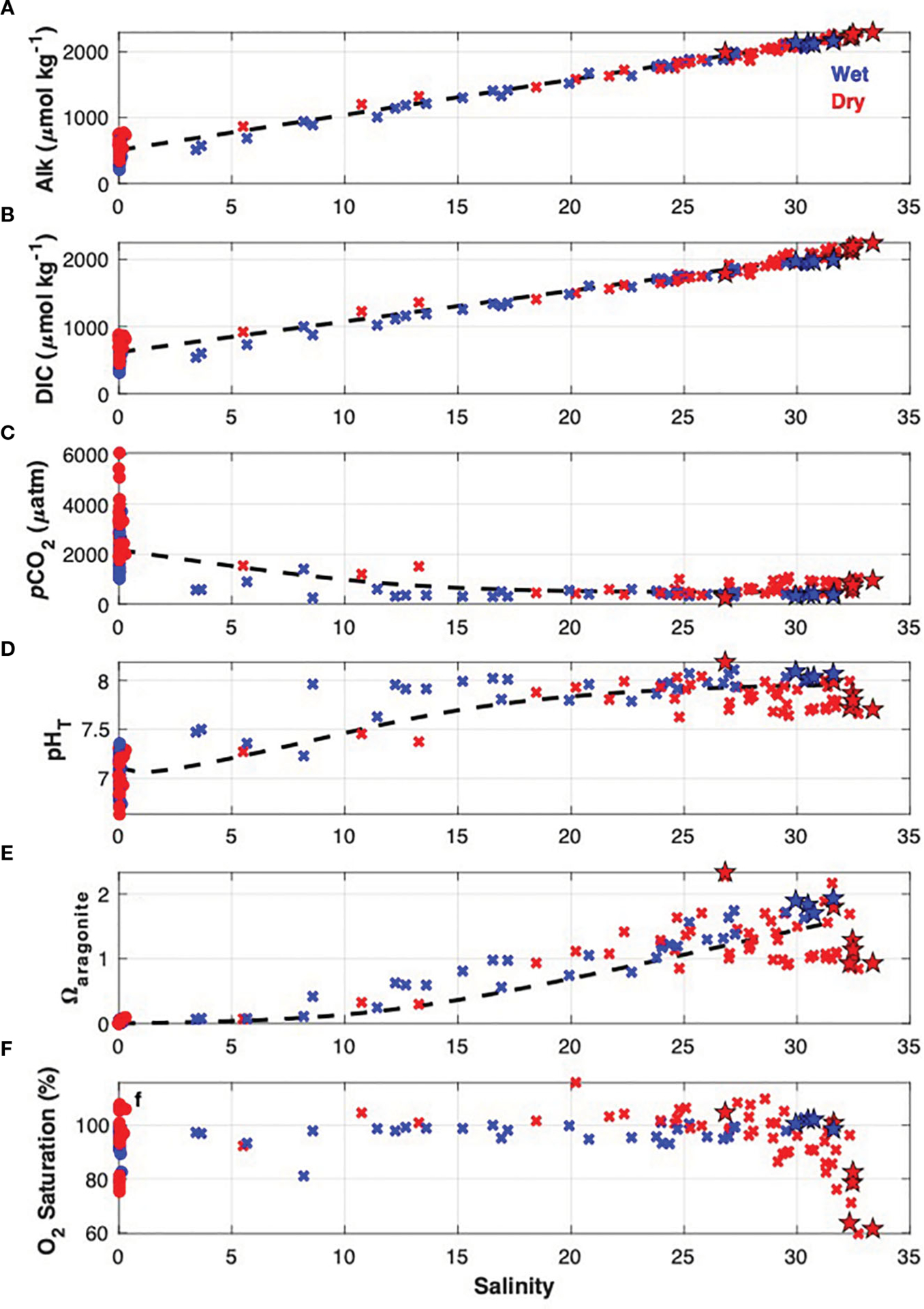
Observations of **(A)** alkalinity, **(B)** DIC, **(C)** $p{\text{CO}}_{2}$, **(D)** ${\text{pH}}_{\text{T}}$, **(E)** $\Omega_{\text{arag}}$, and **(F)** dissolved oxygen saturation from estuarine stations (“x” markers), river stations (circle markers), and station M (star markers) in Tillamook Bay during the 2017 and 2018 synoptic surveys. Observations are color-coded by wet (blue) and dry (red) seasons. Dashed lines indicate the reference mixing lines (REML) between average coastal ocean and river end-members, as defined in Methods section.

Coastal ocean waters entering Tillamook Bay sampled at station “M” during July and August surveys displayed high $p{\text{CO}}_{2}$, low ${\text{pH}}_{\text{T}}$, low $\Omega_{\text{arag}}$, low ${\text{O}}_{2}$, and cold temperatures consistent with seasonally upwelled waters of the northern California Current (Figure 5, Supplementary Table S2; Hales et al., 2005; Chan et al., 2017). Ocean salinity, DIC, and Alk were highest during the dry season, lower in the wet season, and lowest in the spring transition period (Figure 5, Supplementary Table S2). Ocean waters entering the estuary during the wet season surveys were higher in ${\text{O}}_{2}$ and ${\text{pH}}_{\text{T}}$ when compared with the dry season - likely due to the absence of upwelling in the wet season. $\Omega_{\text{arag}}$ during July and August ranged from 0.9 – 1.3, with wet season values near 2 (Figure 5E). $p{\text{CO}}_{2}$ values indicated nearshore ocean waters were a source of ${\text{CO}}_{2}$ to the atmosphere in July, August, and September, near atmospheric levels in October and January, and a sink in February, March, and May (Figure 5C, Supplementary Table S2).

**FIGURE 6:**
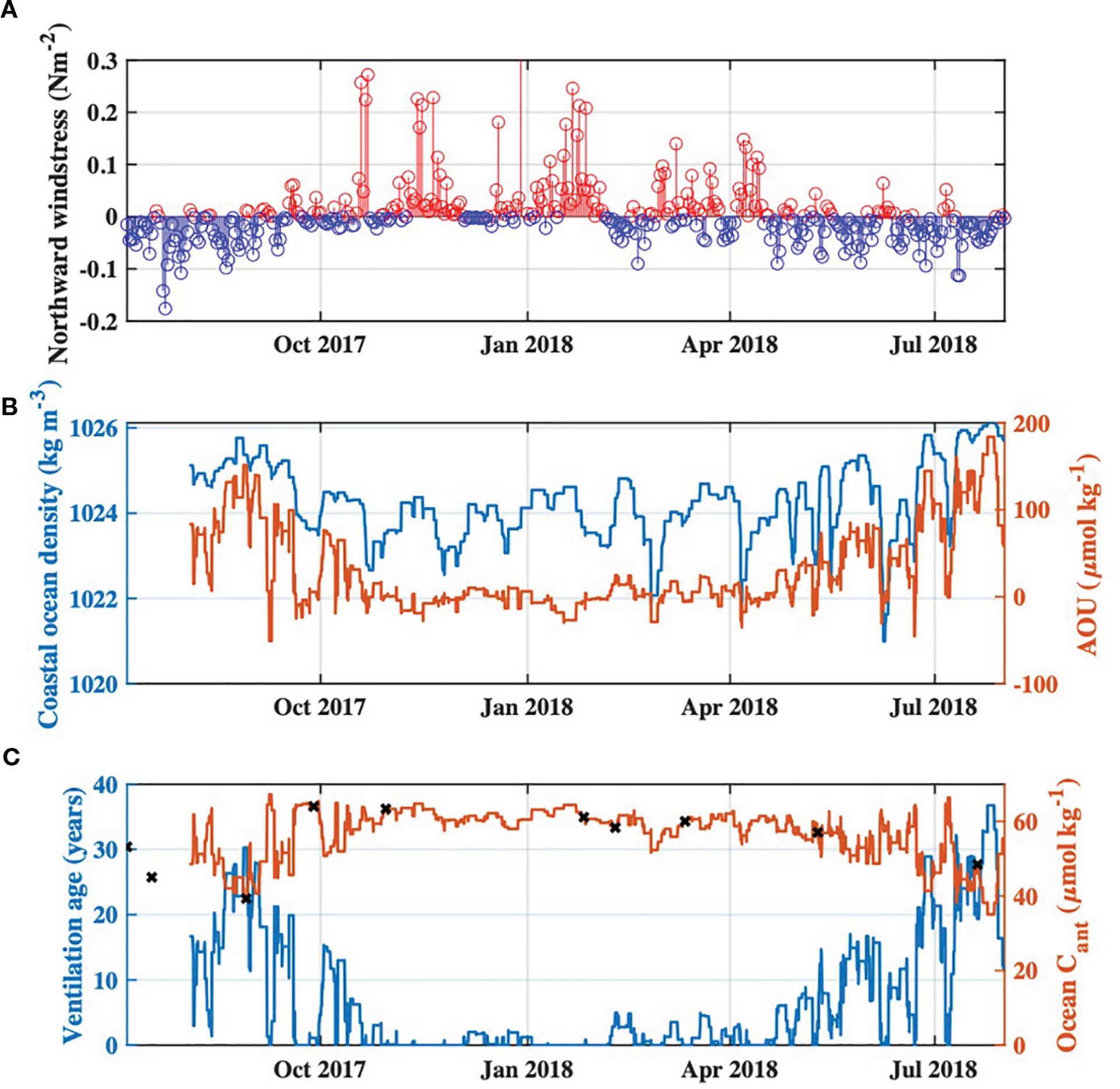
Time series of **(A)** Northward wind stress during the study period from http://damp.coas.oregonstate.edu/windstress/index.html, with positive values indicated by red and negative values indicated by blue, **(B)** Time series of 3-day moving maximum water density, and associated apparent oxygen utilization (AOU), at the Garibaldi Dock monitoring site for the study period, and **(C)** Calculated ventilation age of coastal ocean end-member waters entering Tillamook Estuary, and associated ${\text{C}}_{\text{ant}}$ concentrations. ${\text{C}}_{\text{ant}}$ values specific to each of the synoptic surveys are indicated by the black “x” markers.

Observed AOU in coastal ocean waters entering Tillamook Bay was highest during the summers of 2017 and 2018, and co-occurred with relatively dense waters entering the estuary typical of coastal upwelling. Estimated ventilation ages of coastal ocean waters during these summer periods were relatively high, ranging from ~20–35 years, which agreed reasonably well with a previously published ventilation age of 25 years for upwelling source waters from the California Undercurrent (Murray et al., 2015). The temporal dynamics of estimated ventilation ages tracked observed northward wind stress during the study period (Figure 6A), with higher ventilation ages during periods of upwelling-favorable winds (summer) and lower ventilation ages during times of relaxation or downwelling favorable winds (wet season; Figures 6A–C). Wind stress was not used to calculate ventilation age nor ${\text{C}}_{\text{ant}}$, and therefore served as an independent check on the ability of our calculations to reproduce reasonable temporal dynamics of upwelling influences on ${\text{C}}_{\text{ant}}$ and ventilation ages of coastal ocean waters. Estimated ${\text{C}}_{\text{ant}}$ ranged from 35 to $67\;\mu \text{mol}\;{\text{kg}}^{-1}$, with an average value of $56\;\mu \text{mol}\;{\text{kg}}^{-1}$ (Figure 6C) and was in agreement with previously published values of 37 to $57\;\mu \text{mol}\;{\text{kg}}^{-1}$ for upwelling source waters (Feely et al., 2016; Chan et al., 2017) and 37 to $60\;\mu \text{mol}\;{\text{kg}}^{-1}$ for nearshore surface waters in the region (Feely et al., 2016; Hare et al., 2020). ${\text{C}}_{\text{ant}}$ was estimated to be lowest during periods of summer upwelling due to the older ventilation ages of upwelled coastal ocean waters, thus last being in contact with lower atmospheric ${\text{CO}}_{2}$ levels (Figures 6B, C). ${\text{C}}_{\text{ant}}$ was highest during the wet season when AOU was low (or positive) and ventilation ages were therefore estimated to also be low or zero (i.e. equilibrated with modern atmospheric ${\text{CO}}_{2}$ levels) (Figures 6B, C). This behavior of ${\text{C}}_{\text{ant}}$ with respect to upwelling dynamics is in agreement with observations of both Feely et al. (2016) and Chan et al. (2017).

#### 3.1.3 Seasonal observations of Tillamook Bay carbonate chemistry

${\text{pH}}_{\text{T}},\Omega_{\text{arag}}$, and $p{\text{CO}}_{2}$ observations at the estuarine synoptic survey stations largely followed patterns expected from seasonal variabilities of end-member carbonate chemistry and river discharge (Figure 5). The lowest ${\text{pH}}_{\text{T}}$, lowest $\Omega_{\text{arag}}$, and highest $p{\text{CO}}_{2}$ observations during this study in polyhaline portions of the estuary occurred during the July and August surveys, coinciding with similar patterns in coastal ocean chemistry. Estuary stations during these dry season surveys had a mean ${\text{pH}}_{\text{T}}$ of 7.77, mean $\Omega_{\text{arag}}$ of 1.10, and mean $p{\text{CO}}_{2}$ of 785μatm. September and October surveys were influenced by increasing river discharge (Figures 4, 5, Supplementary Table S2) and lower ${\text{CO}}_{2}$ (higher ${\text{pH}}_{\text{T}}$ and $\Omega_{\text{arag}}$) coastal ocean conditions, with observed estuarine station mean ${\text{pH}}_{\text{T}},\Omega_{\text{arag}}$, and $p{\text{CO}}_{2}$ of 7.86, 1.31, and 602μatm, respectively. January, February, and March surveys occurred during periods of relatively high river discharge and lower ${\text{CO}}_{2}$ (higher ${\text{pH}}_{\text{T}}$ and $\Omega_{\text{arag}}$) coastal ocean conditions similar to the fall, with observed estuarine station mean ${\text{pH}}_{\text{T}},\Omega_{\text{arag}}$, and $p{\text{CO}}_{2}$ of 7.81, 0.90, and 586μatm, respectively. The May survey appeared to be moderately influenced by river discharge, similar to the fall surveys, with observed estuarine station mean ${\text{pH}}_{\text{T}},\Omega_{\text{arag,}}$ and $p{\text{CO}}_{2}$ of 7.90, 1.34, and 581μatm, respectively.

**FIGURE 7:**
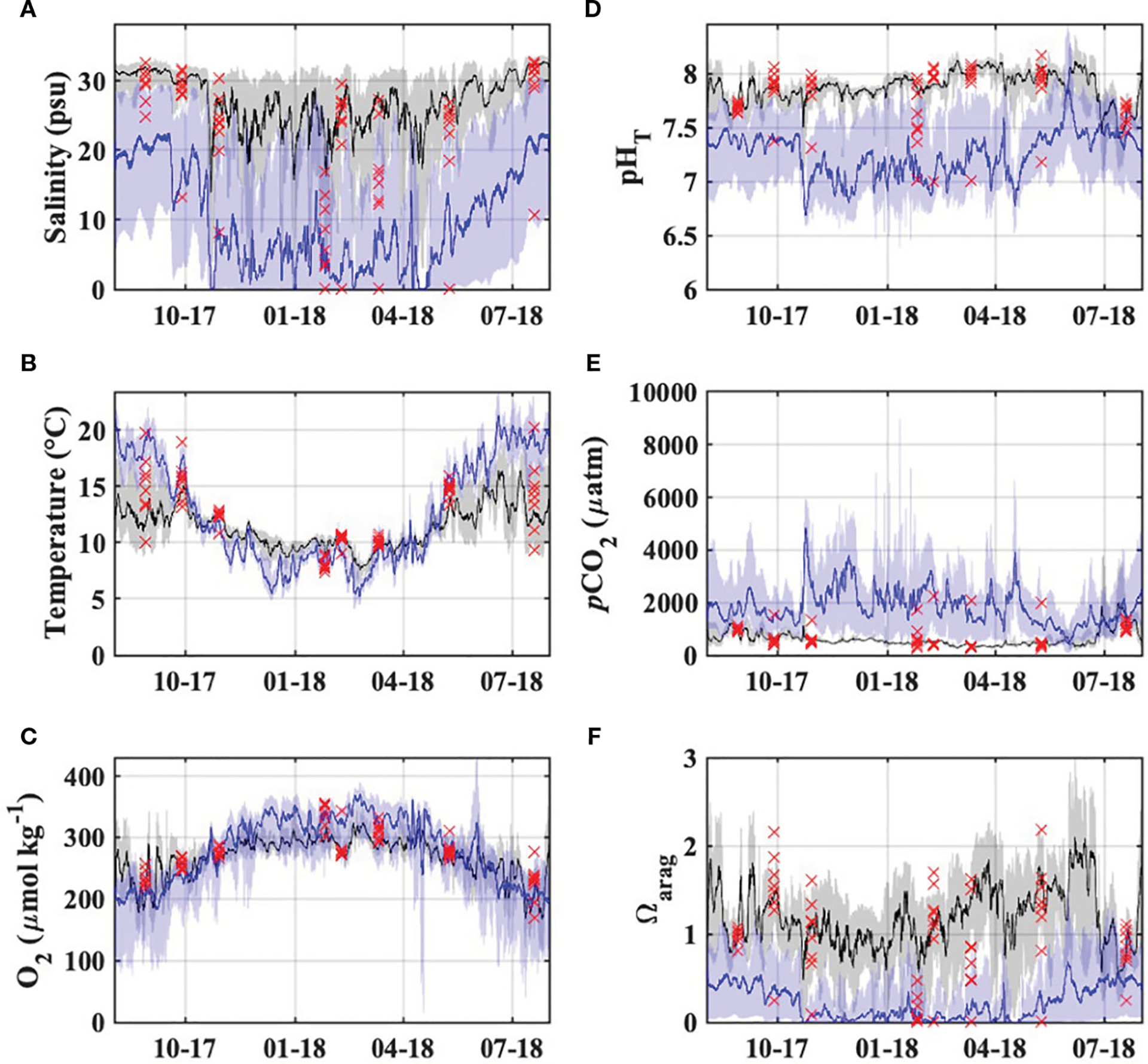
Observed **(A)** salinity, **(B)** temperature, **(C)** dissolved oxygen saturation, and **(D-F)** carbonate chemistry in the Tillamook Estuary from the synoptic surveys (red markers), Garibaldi Dock monitoring (black) and TB-01 monitoring (blue). Lines and shaded regions represent daily moving averages and full observed ranges, respectively.

Monitoring at the Garibaldi Dock and TB-01 stations provided continuous observations of biogeochemical variability within Tillamook Bay which complement the high-quality discrete observations from the synoptic estuary surveys (Figure 7). Observed ${\text{pH}}_{\text{T}}$, along with calculated $\Omega_{\text{arag}}$ and $p{\text{CO}}_{2}$, were highly variable on diel, event, and seasonal time scales. Seasonal carbonate chemistry dynamics at the sites showed opposing patterns; the Garibaldi Dock station experienced lower ${\text{pH}}_{\text{T}}$ and higher ${\text{pCO}}_{2}$ during periods of low river discharge and upwelling-favorable conditions in the dry season, with highly variable $\Omega_{\text{aras}}$ throughout the year, while the TB-01 station experienced lower ${\text{pH}}_{\text{T}}$ and $\Omega_{\text{arag}}$, with higher $p{\text{CO}}_{2}$, during periods of high river discharge in the wet season (Figure 7).

The lowest ${\text{pH}}_{\text{T}}$ and $\Omega_{\text{arag}}$ observations at the Garibaldi Dock were associated with short-duration events in the fall, winter, and spring associated with low salinities (<10) that were coincident with spikes in river discharge and northward winds often associated with low pressure systems and precipitation events (Figure 7, Supplementary Figure S9; Bane et al., 2005), and longer duration events in the summer of 2018 associated with high salinity (>32), low temperatures (< 12°C), and low dissolved oxygen characteristic of freshly upwelled coastal ocean waters with a large metabolic ${\text{CO}}_{2}$ signal. Both of these environmental conditions were also characterized by high $\left(>1000\;\mu \text{atm}\right)\;p{\text{CO}}_{2}$ values. During the study period, $p{\text{CO}}_{2}$ values exceeded 410μatm (i.e. modern atmospheric values) for 75% of observations, with the highest values during summer 2018 upwelling events. Waters at Garibaldi were undersaturated with respect to $\Omega_{\text{arag}}$ (<1.0 units) in 35% of the observations, and only exceeded 1.5 units for 26% of the observed period. Discrete observations of ${\text{pH}}_{\text{T}},\Omega_{\text{arag}}$, and $p{\text{CO}}_{2}$ from the estuary surveys showed generally good agreement with the Garibaldi mooring observations at comparable salinities (Figure 7)

The location of TB-01 in the oligo/mesohaline portion of the estuary, with stronger river discharge influence, resulted in lower salinity waters at the site. These waters were generally of lower ${\text{pH}}_{\text{T}}$ and $\Omega_{\text{arag,}}$, and higher $p{\text{CO}}_{2}$, when compared with Garibaldi, and displayed more seasonal temperature and dissolved oxygen variability (Figure 7). Waters were nearly always undersaturated with respect to $\Omega_{\text{arag}}$ (99% of observations), and a strong source of ${\text{CO}}_{2}$ to the atmosphere (99% of observations >410μatm). Diel variability of ${\text{pH}}_{\text{T}},p{\text{CO}}_{2}$, and $\Omega_{\text{arag}}$ at TB-01 was much higher than at Garibaldi, likely driven by strong tidal mixing and local metabolic processes as evidenced by the high variabilities of salinity and dissolved oxygen saturation.

### 3.2 Mechanistic drivers of Tillamook Bay carbonate chemistry

**FIGURE 8:**
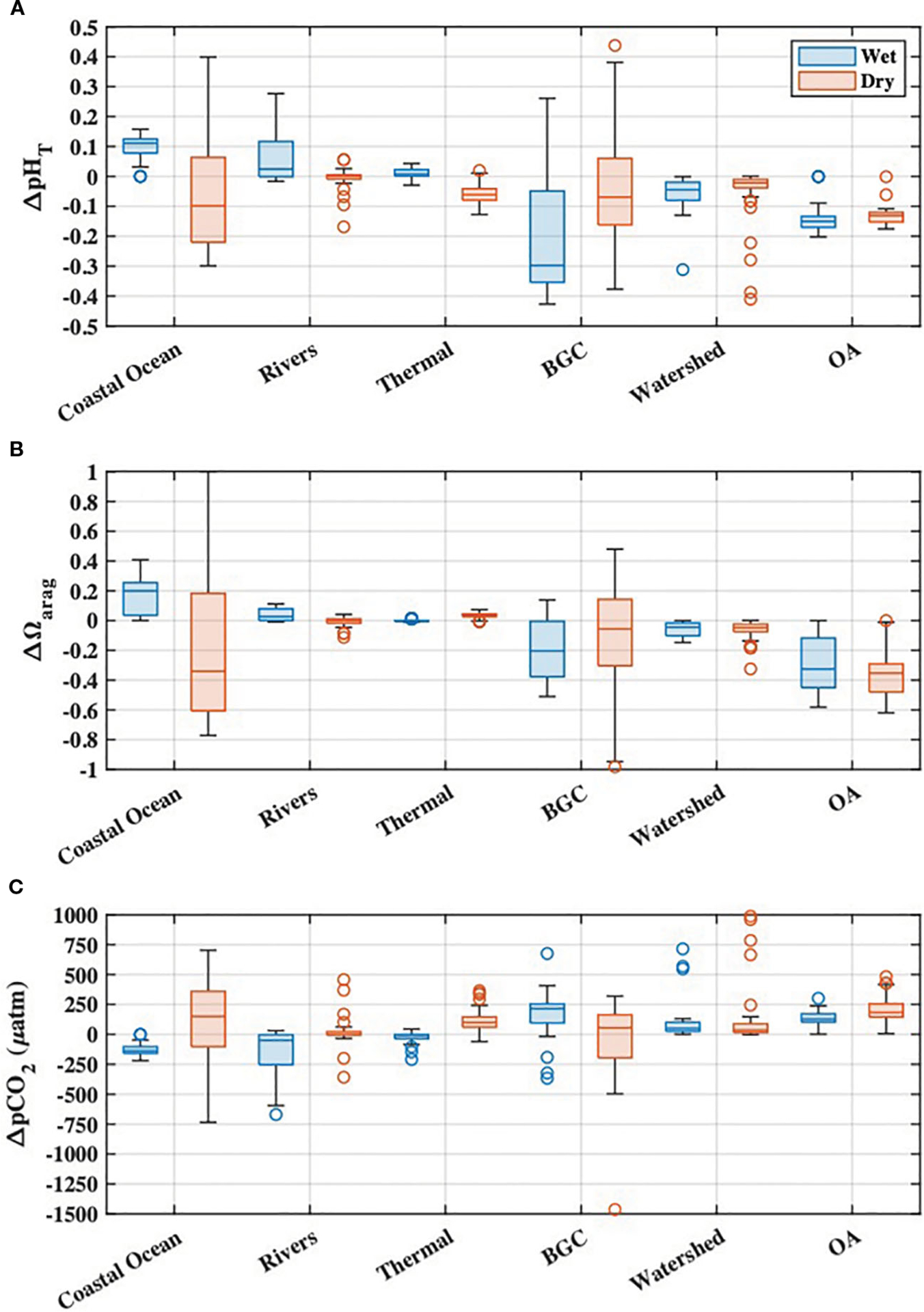
Mechanistic drivers of Tillamook Bay seasonal **(A)** ${\text{pH}}_{\text{T}}$, **(B)** $\Omega_{\text{arag}}$, and **(C)** ${p\text{CO}}_{2}$ calculated from synoptic survey observations. Impacts of riverine carbon enrichments (“Watershed”) and ocean acidification (“OA”) on estuarine chemistry are also shown.

The first-order variability of Tillamook Bay carbonate chemistry was driven by mixing between the coastal ocean and river end-members, evidenced by the close adherence of DIC and Alk to the reference mixing lines for each parameter (Figure 5). OA was found to drive larger decreases in $\Omega_{\text{arag}}$ during both wet and dry seasons when compared with all processes responsible for departures of estuarine ${\text{pH}}_{\text{T}},\Omega_{\text{arag}}$, and $p{\text{CO}}_{2}$ from reference mixing line values (i.e. the variabilities after accounting for conservative mixing) (Figures 8A–C). BGC impacts to estuarine chemistry were the most variable with respect to both magnitude and sign, but generally lowered ${\text{pH}}_{\text{T}}$ and $\Omega_{\text{arag}}$ while raising $p{\text{CO}}_{2}$ during the wet and dry seasons. The most frequent exceptions to this pattern occurred during the dry season, where apparent autotrophy (evidenced by ${\text{O}}_{2}$ supersaturation; Figure 7C) drove increases in estuarine ${\text{pH}}_{\text{T}}$ and $\Omega_{\text{arag}}$ while lowering $p{\text{CO}}_{2}$. Coastal ocean dynamics contributed to relatively higher ${\text{pH}}_{\text{T}}$ and $\Omega_{\text{arag}}$, and lower $p{\text{CO}}_{2}$ during the wet season, while upwelling during the dry season caused relatively large decreases of estuarine ${\text{pH}}_{\text{T}}$ and $\Omega_{\text{arag,}}$ and increased $p{\text{CO}}_{2}$. The majority of river influence on estuarine chemistry was via direct dilution of coastal ocean waters (Figure 5), with seasonal variability of the river end-member responsible for relatively small changes to estuarine carbonate chemistry (Figure 8). We note that seasonal variability in the ocean and river end-members implicitly includes the variability of anthropogenic signals in these same source waters, as characterized by ${\text{C}}_{\text{ant}},{\text{DIC}}_{\text{enrich}}$, and ${\text{Alk}}_{\text{enrich}}$. Thermal effects on estuarine chemistry were also small, with the warming during the dry season causing decreased ${\text{pH}}_{\text{T}}$, and increased $\Omega_{\text{arag}}$ and $p{\text{CO}}_{2}$. Both riverine carbon enrichments and OA reduced estuarine ${\text{pH}}_{\text{T}}$ and $\Omega_{\text{arag}}$, and raised $p{\text{CO}}_{2}$, during the wet and dry seasons (Figure 8). Seasonal OA impacts to estuarine carbonate chemistry were always larger than the riverine carbon enrichment impacts, and similar in magnitude to coastal ocean variability and local BGC variability.

### 3.3 Anthropogenic impacts to Tillamook Bay carbonate chemistry from watershed and coastal ocean influences

**FIGURE 9:**
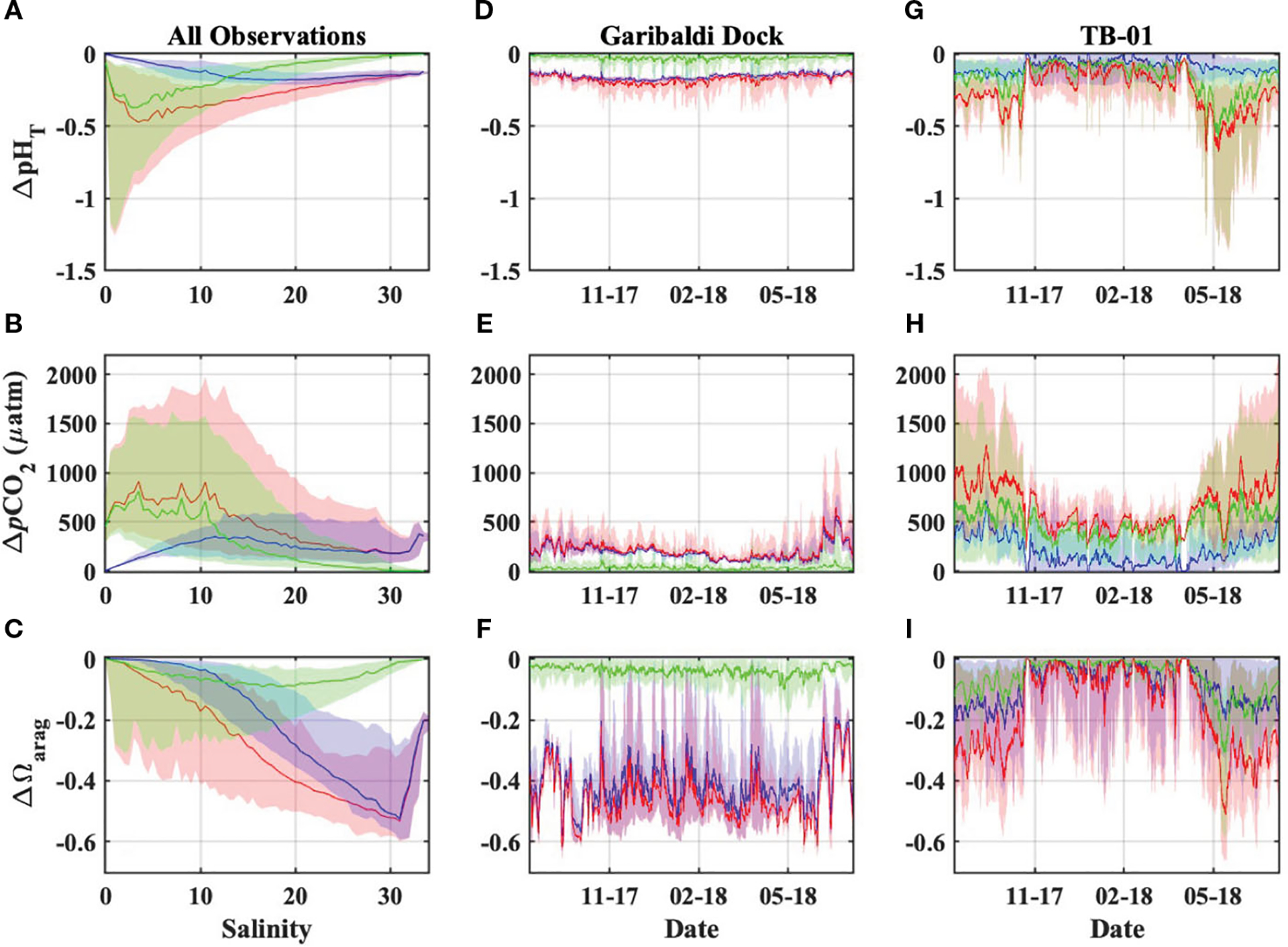
Estimated impacts to Tillamook Estuary carbonate chemistry due to OA (blue), riverine carbon enrichments (green), and the combination of both OA and riverine carbon enrichments (red). These impacts are presented as they vary **(A-C)** pseudo-spatially across the observed estuarine salinity spectrum using observations from both monitoring stations, and **(D-I)** temporally using observations from the Garibaldi Dock and TB-01 monitoring stations. Plots **(A-C)** display the medians (lines) and 90% inter-quantile ranges (shaded area) of water quality impacts.

**FIGURE 10:**
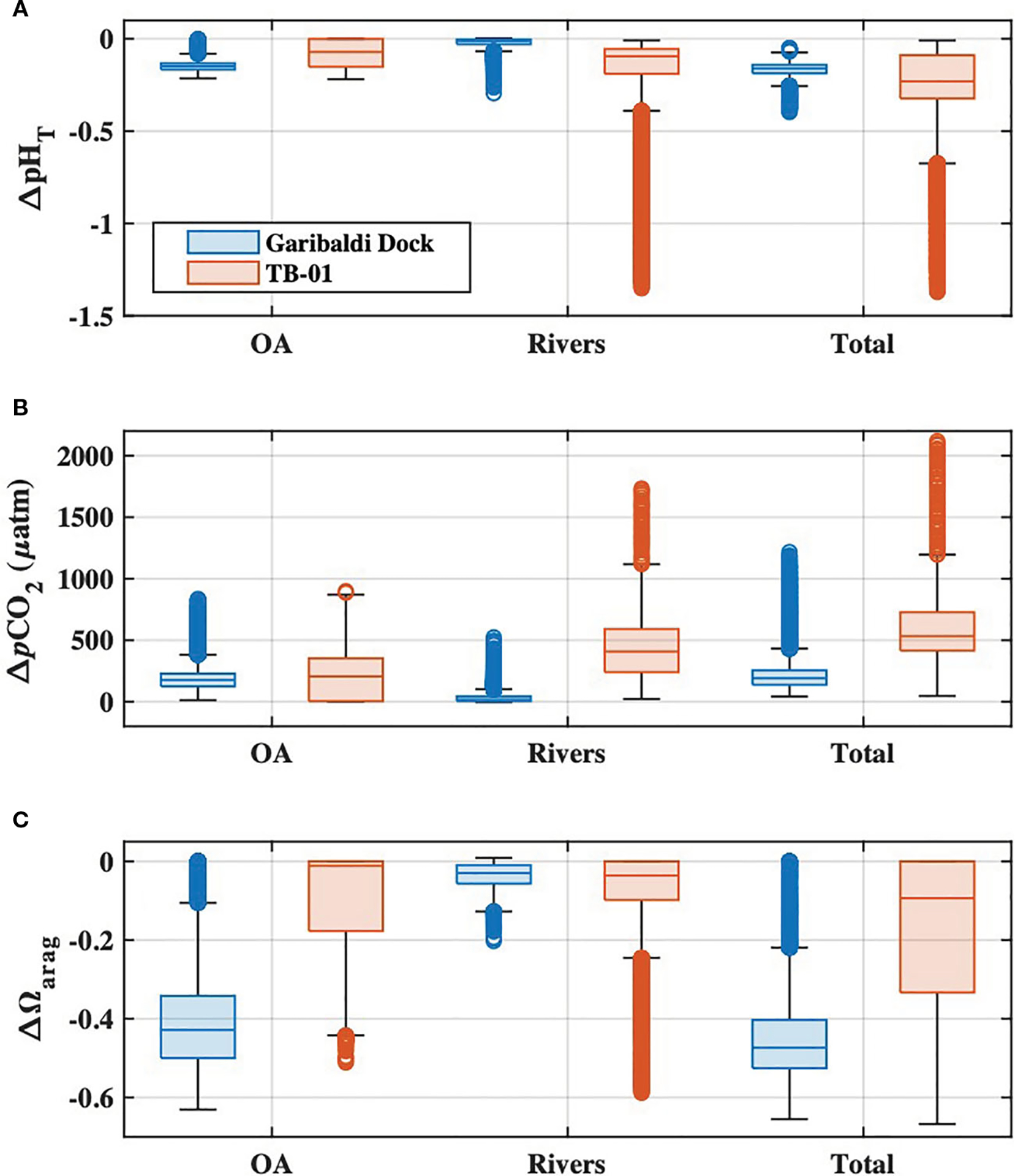
Boxplots of **(A)** pH_T_, **(B)**, ${p\text{CO}}_{2}$, and **(C) $\Omega_{\text{arag}}$** impacts at the Garibaldi Dock (blue) and TB-01 (red) monitoring sites due to ocean acidification (‘OA’) riverine carbon enrichments (‘Rivers’), and the combination of both drivers (‘Total’). Shaded regions of each boxplot represent the median and interquartile range, whiskers represent the full range of data (minus outliers), and the open circles represent outlier values.

OA was the dominant driver of estuarine ${\text{pH}}_{\text{T}}$ and $\Omega_{\text{arag}}$ declines and $p{\text{CO}}_{2}$ increases, at salinities greater than ~13, below which riverine carbon enrichments became more important for coastal acidification impacts (Figures 9A–C). The present-day combined impacts of ocean acidification and riverine carbon enrichments on estuarine water quality were highly variable in both space and time, and always greater than those impacts from either driver alone (Figure 9). Declines in estuarine ${\text{pH}}_{\text{T}}$ were greatest at low salinities (sometimes exceeding one full pH unit) and highly variable (ranging from ~0 to 1.4 units), while polyhaline portions of the bay experienced more moderate and consistent declines of ~0.1 to ~0.2 units (Figures 9A, D, G). Changes to estuarine $p{\text{CO}}_{2}$ were generally variable, with the largest increases at low and moderate salinities (Figures 9B, E, H). The largest $\Omega_{\text{arag}}$ declines occurred in polyhaline portions of the estuary (Figures 9C, F, I) and were primarily driven by ocean acidification. $\Omega_{\text{arag}}$ was generally undersaturated (< 1) in oligo- and mesohaline portions of the estuary (Figures 5E, 7F), and decreases in these waters were therefore relatively small when compared with $\Omega_{\text{arag}}$ decreases at higher salinities. Overall, the total impacts of ocean acidification and riverine carbon enrichments at the Garibaldi Dock reduced ${\text{pH}}_{\text{T}}$ by 0.16 units, increased $p{\text{CO}}_{2}$ by 192μatm, and reduced median $\Omega_{\text{arag}}$ by 0.47 units (Figures 9A–C). Total impacts at TB-01 reduced ${\text{pH}}_{\text{T}}$ by 0.23 units, increased $p{\text{CO}}_{2}$ by 533μatm, and reduced median $\Omega_{\text{arag}}$ by 0.1 units (Figures 10A–C). Riverine carbon enrichments were the dominant driver of $\Delta {\text{pH}}_{\text{T}}$ and $\Delta p{\text{CO}}_{2}$ at TB-01, while OA was generally less important for $\Delta \Omega_{\text{arag.}}$. Impacts of riverine carbon enrichments were relatively small year-round at the Garibaldi Dock location, and only episodically important during periods of elevated river discharge (Figures 9, 10).

**FIGURE 11:**
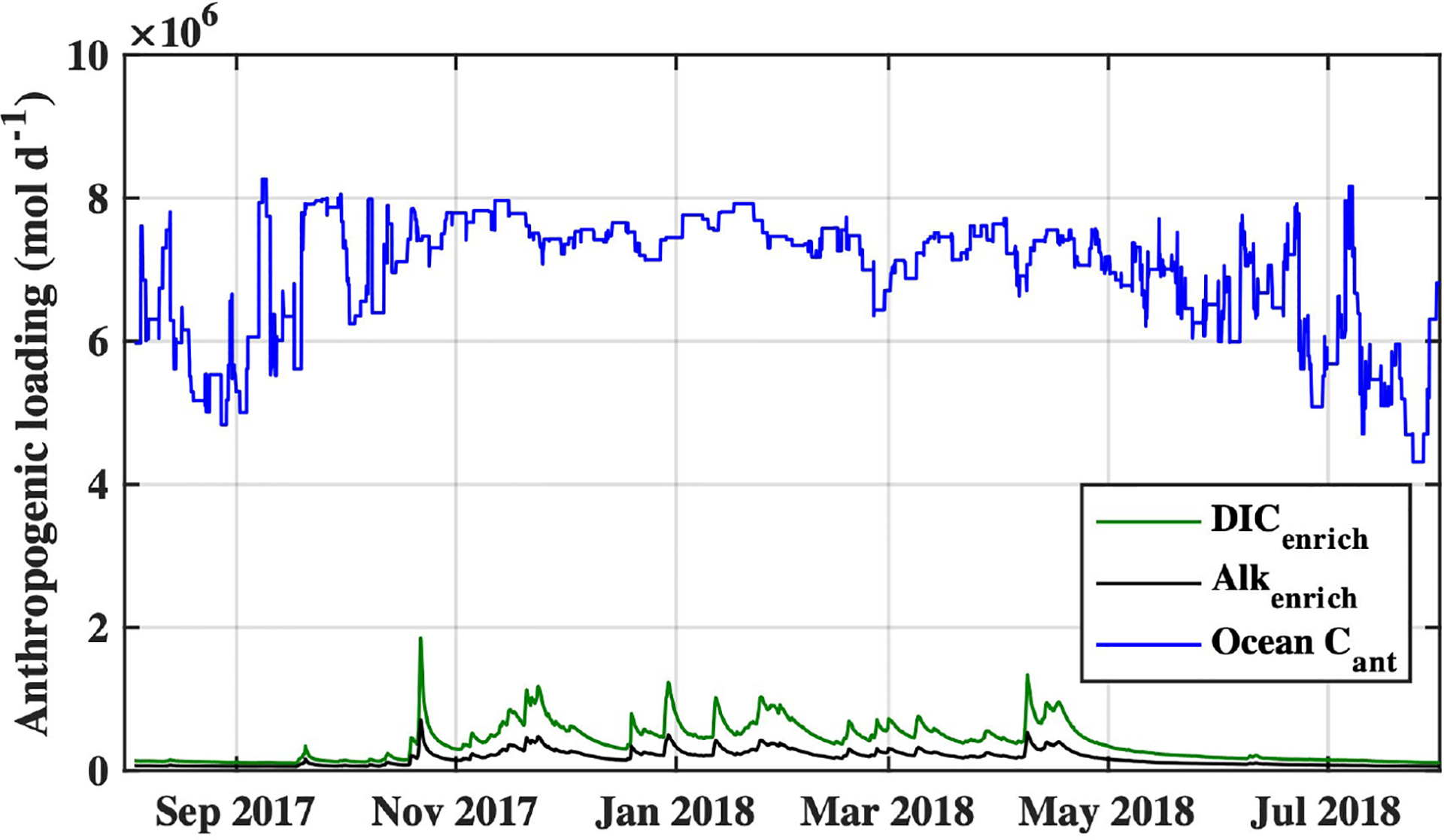
Estimates of anthropogenic carbon loading to Tillamook Bay from coastal ocean ${\text{C}}_{\text{ant}}$ (blue), and riverine enrichments of DIC (green) and Alk (black) during the study period.

At the whole-estuary scale, anthropogenic carbon loading was dominated by tidal exchange of coastal ocean $C_{\text{ant}}$, with< 6% of the loading coming from riverine carbon enrichments on average (Figure 11). Average ocean ${\text{C}}_{\text{ant}}$ loading during the study period was $6.9\times 10^{6}\text{mol}\;{\text{d}}^{-1}$, while loading from ${\text{DIC}}_{\text{enrich}}$ and ${\text{Alk}}_{\text{enrich}}$ averaged $3.7\times 10^{5}\text{mol}\;{\text{d}}^{-1}$ and $1.7\times 10^{5}\text{mol}\;{\text{d}}^{-1}$, respectively. Anthropogenic carbon loading to the estuary was greatest during the wet season, coincident with high river discharge and high coastal ocean $C_{\text{ant}}$ concentrations, and lowest during the dry season during upwelling periods and low river discharge.

## 4 Discussion

### 4.1 Coastal upwelling influences on Tillamook Bay carbonate chemistry

Previous studies in the California Current have shown that coastal upwelling can strongly control shelf (Hales et al., 2005; Feely et al., 2008; Harris et al., 2013) and estuarine (Barton et al., 2012; Hales et al., 2017; Fairchild and Hales, 2021) carbonate chemistry dynamics. Our study characterizes the dominant role of coastal ocean conditions in the anthropogenic acidification dynamics of Tillamook Bay, especially in the polyhaline lower estuary. The many low residence time estuaries in the Northeast Pacific are subject to similar oceanic influence (Hickey and Banas, 2003), thus creating concerns from a water quality perspective given that the lowest ${\text{pH}}_{\text{T}}$ and most corrosive upwelled waters occur in the most nearshore regions of the shelf (Feely et al., 2016). Estuarine ecosystems are therefore naturally exposed to some of the most extreme carbonate chemistry conditions experienced in the California Current system, even prior to any addition of metabolic carbon generated from estuarine net community metabolism. Freshly upwelled waters also have high DIC : Alk ratios and high sensitivity to further acidification, contributing to these systems being more vulnerable to local acidification and corrosive conditions (Cai et al., 2021) than open-ocean environments. While estuarine ecosystems have likely always experienced high natural variability and biologicallystressful conditions (Hales et al., 2017), periods of active upwelling may therefore be especially prone to accelerated anthropogenic acidification (Chan et al., 2017; Feely et al., 2018) on top of an already stressful natural baseline. Estuarine water quality during upwelling periods has already been shown to significantly impair Pacific oyster hatchery operations attributable to lower $\Omega_{\text{arag}}$ (Barton et al., 2012), and upwelling events for this region are predicted to become stronger and longer in duration with ongoing climate change (Iles et al., 2012). Additionally, changes in the relative contributions of North Pacific and Equatorial Pacific waters to the California Current over the past 30 years have been shown to result in more acidified source waters (Meinvielle and Johnson, 2013), which are delivered to coastal and estuarine habitats during upwelling events. The interannual and decadal variability of future coastal acidification in California Current coastal and estuarine habitats will likely be significantly modulated by these coastal ocean upwelling dynamics, and increasingly worse due to atmospheric ${\text{CO}}_{2}$ trends. Interpretation of long-term carbonate system changes in estuarine habitats would benefit from accounting for these relevant local upwelling dynamics (Turi et al., 2016).

### 4.2 Anthropogenic impacts to riverine water quality in the Tillamook Bay watershed

Given the literature demonstrating anthropogenic enrichment of river DIC consistent with our observations, and the concurrent increases of downriver DIC with both ${{\text{NO}}_{3}}^{-}$ levels and indices of human land use observed in our study, this evidence taken together suggests human activities in the watershed are responsible for some portion of the observed downriver changes in riverine carbonate chemistry. A concurrent study of nitrate isotopes and bacterial source tracking in the Tillamook watershed showed significant enrichment of $\delta^{15}\text{N-}{\text{NO}}_{3}$ and markers for septic and ruminant bacteria at the same downriver sampling locations where we observed enriched DIC and ${\text{NO}}_{3}$, and reduced $\text{p}{\text{H}}_{\text{T}}$ (A.G. Zimmer-Faust et al., submitted to *Water Research*). We are unaware of other studies characterizing anthropogenic land use effects on the carbonate chemistry of coastal rivers discharging to the California Current, but previous work has shown that DIC content of temperate streams is strongly related to watershed land use (Barnes and Raymond, 2009; Zhang et al., 2009; Kaushal et al., 2017). The strong negative relationship we observed between river discharge and downriver enrichments of DIC and Alk (Supplementary Figure S4) suggests dilution of a downriver source in the areas with increased human activity. Tillamook County contains 36,551 acres of farmland, 46% of which are treated with commercial fertilizers, lime and soil conditioners, and manure (USDA-NASS, 2012) which can affect river chemistry. While these numbers are county-wide, much of this agricultural activity is concentrated in the lower Tillamook Bay watershed captured by our study sites (Figure 1). The bacterial contamination issues previously documented in the estuary related to agricultural runoff, septic system discharges, and wastewater discharges also demonstrate a direct link between anthropogenic land use in the watershed and water quality in the estuary. It therefore seems likely that other water quality indices in Tillamook Bay, including ${\text{pH}}_{\text{T}}$ and other carbonate parameters measured in this study, are affected by these same land use and development practices. The majority of observed downriver DIC additions were in the form of ${{\text{HCO}}_{3}}^{-}$ with smaller additions of ${\text{CO}}_{2(\text{aq})}$, causing downriver DIC and Alk additions to nearly always fall above a 1:1 DIC to Alk ratio line (Supplementary Figure S8). The addition of ${{\text{HCO}}_{3}}^{-}$ in temperate streams and rivers has generally been interpreted as resulting from weathering of carbonate and silicate minerals (Oh and Raymond, 2006; Barnes and Raymond, 2009; Joesoef et al., 2017), but has been shown to be anthropogenically enhanced by lime additions and acids produced by metabolic processing of agricultural fertilizers and manure (Oh and Raymond, 2006). The downriver DIC and Alk additions in the Tillamook watershed (primarily ${{\text{HCO}}_{3}}^{-}$ with smaller amounts of ${\text{CO}}_{2(\text{aq})}$) would be consistent with the delivery of anerobic and aerobic metabolites to the lower rivers from areas with increased human land use, and is supported by the observations of elevated ${{\text{NO}}_{3}}^{-}$ concentrations at downstream stations. The downriver DIC additions could also be explained by carbonate dissolution in the lower watershed, with co-occurring additions of metabolic ${\text{CO}}_{2\text{(aq)}}$ and/or organic acids. However, much of the lower Tillamook watershed consists of flood plains which may naturally deliver higher DIC and Alk loads via degradation of accumulated organic matter-rich soils to the rivers when compared with the upper forested watersheds. Attributing the totality of observed river water quality changes to specific anthropogenic mechanisms in the Tillamook watershed is not possible with the current dataset and is a topic of ongoing study. We therefore posit that the observed downriver changes in riverine carbonate chemistry represent upper bounds of the human influence on river chemistry in the Tillamook Bay watershed during our study, and are useful for understanding potential land-based impacts on the acidification dynamics of the estuary as discussed below. The land-based impacts presented in this study are, however, limited to direct impacts to riverine water quality (and resultant downstream changes in estuarine water quality), and further study is necessary to understand if autochthonous processing of anthropogenic nutrients and organic matter delivered by these waters is a significant driver of estuarine water quality impacts. Despite the short residence time of waters in Tillamook Bay, local biogeochemical processes were an important driver of observed carbonate chemistry (Figure 8) and suggest that anthropogenic modification of local metabolism could have an appreciable impact on estuarine water quality.

### 4.3 Interacting acidification drivers in Tillamook Bay and broader implications

As our understanding of the complexity of acidification in estuarine systems continues to improve, accounting for changes to land-based delivery of carbon and alkalinity is necessary to accurately characterize water quality impacts in these systems This is especially important, considering that altered river delivery of DIC and Alk have been shown to both improve and degrade water quality indicators of acidification (Van Dam and Wang, 2019; Da et al., 2021). Tillamook Bay is an example of contrasting perspectives on acidification impacts in an estuary: riverine-driven acidification was responsible for the most severe acidification impacts to water quality, but these were limited in spatial scope and the OA signal dominated acidification impacts at the whole-system scale. Acidification effects in Tillamook Bay were also spatially and temporally heterogeneous, and demonstrate the complexity of acidification dynamics in estuarine environments.

The importance of riverine carbon enrichments to acidification impacts in lower-salinity areas, and the temporal variability of ocean ${\text{C}}_{\text{ant}}$ entering the estuary due to seasonal upwelling, highlight the potential pitfalls of assuming estuarine waters will track atmospheric ${\text{CO}}_{2}$ trajectories (Carstensen et al., 2018; Carstensen and Duarte, 2019; Cai et al., 2021). While water quality impacts in polyhaline waters of Tillamook Bay were dominated by OA, these impacts were greater than would be assumed in well-buffered ocean waters and were still influenced by enhanced riverine carbon delivery. Critically, the water quality impacts of riverine carbon enrichments alone in Tillamook Bay were shown to be as large, or larger, than those changes driven by OA in moderate and low salinity waters, with the combined effects of the two drivers being additive. Enhanced rates of acidification and water quality degradation in estuaries have been long known (Cai et al., 2011; Waldbusser et al., 2011; Sunda and Cai, 2012) and more recently shown to hasten the exceedance of water quality and organismal tolerance thresholds (Pacella et al., 2018; Cai et al., 2021). Our study builds upon this work by providing a methodological framework for characterizing the temporal and spatial dynamics of estuary acidification at scales relevant for endemic organisms (e.g. Bednarsek et al., 2019).

Despite the dominant role of OA to water quality impacts in Tillamook Bay, the variance in rates of acidification amongst estuaries (and susceptibility to changes in water quality) more generally is likely to be primarily determined by local watershed chemistry and biophysical (e.g. metabolic rates, estuarine residence and freshwater flushing times, stratification) drivers. While there is variability in oceanic ${\text{C}}_{\text{ant}}$ in coastal zones of the United States (Feely et al., 2018), this is often smaller than the anthropogenic changes to riverine DIC and Alk delivery in anthropogenically-altered watersheds studied to date (Raymond et al., 2008; Stets et al., 2014; Da et al., 2021). Acidification impacts in estuaries are modulated by the buffering characteristics of estuarine waters, and are a function of the DIC and Alk concentrations of the watershed end-member (see Cai et al., 2021 for a discussion). Further study is necessary to more broadly understand the characteristics of coastal systems that modulate responses to eutrophication and other land-based acidification drivers, and determine the vulnerability of these systems to coastal acidification.

Our methodology provides a transferable framework for parsing out the anthropogenic versus natural dynamics of acidification across broad estuarine salinity spectrums in similar systems. Application of the same methodology would be most appropriate in short residence time systems like Tillamook Bay, where end-member mixing is the dominant driver of within-estuary carbonate chemistry conditions. Our methodological framework would likely be inappropriate for application in long residence time estuaries such as the Chesapeake Bay, which are typically controlled by complex within-estuary biogeochemical processes (Shen et al. 2019) and more suited to coupled hydrodynamic-biogeochemical models (e.g. Li et al., 2023).

### 4.4 Implications for water quality management in Tillamook Bay and beyond

Characterizing the balance between river and land-based drivers of acidification versus ocean acidification allows one to look critically at estuarine spatial planning in ways to manage the most immediate impacts on estuarine resources. The scope of the potential efficacy of local management actions will depend upon properly matching these strategies to both the spatial and temporal “footprint” of acidification, as well as the magnitude of water quality impacts. To understand the efficacy of potential management actions and support relevant decision making, it is therefore important to characterize the impacts of acidification drivers that would be targeted by such actions.

OA is the result of anthropogenic ${\text{CO}}_{2}$ emissions to the atmosphere on a global scale and will therefore require a coordinated international effort to reverse effects on ocean chemistry (Strong et al., 2014). In parallel with addressing global ${\text{CO}}_{2}$ emissions, there has been interest expressed in the identification of targeted local management and policy actions which effectively ameliorate acidification impacts to coastal and estuarine water quality (Kelly et al., 2011; Strong et al., 2014; Weisberg et al., 2016). Many of the identified possible management actions for curtailing coastal acidification involve reduction of land-based nutrients and other pollutants associated with human land use and urban development, and in-situ carbon removal via phytoremediation or other “blue carbon” strategies. In Tillamook Bay, ${\text{C}}_{\text{ant}}$ delivery from the coastal ocean was responsible for the vast majority of anthropogenic carbon loading (Figure 11), making OA the greatest driver of acidification impacts at the whole-estuary scale and ultimately tying local water quality to global atmospheric ${\text{CO}}_{2}$ emissions. This is likely true for other estuarine systems with low residence times and large tidal prisms, typical of the dozens of U.S. west coast estuaries with small mountainous river watersheds (Bricker et al., 2007). Considering the relatively small loading of anthropogenic riverine carbon to Tillamook Bay, and constraint of most of these land-based water quality impacts to areas with low salinity, reductions of riverine carbon loading alone will be insufficient for amelioration of acidification impacts in most of the estuary. Local management for acidification impacts to water quality in Tillamook Bay may therefore require active interventions, such as possible phytoremediation and other carbon removal strategies. The efficacy of seagrass phytoremediation in Tillamook may be challenged, however, by the already high anthropogenic carbon loading to the bay when compared to typical temperate seagrass carbon removal rates. Current areal rates of anthropogenic carbon loading to Tillamook Bay are $\sim 202\;\text{mmol C}\;{\text{m}}^{-2}{\text{d}}^{-1}$ on average, higher than typical temperate seagrass carbon removal rates of $\sim 33\;\text{mmol C}\;{\text{m}}^{-2}{\text{d}}^{-1}$ (Duarte et al., 2010) (though there is considerable variation in published seagrass areal net primary production). Studies focused on the role of seagrass as a phytoremediation strategy have found seagrasses can raise estuarine pH on the order of ~0.04 units (Koweek et al., 2018) to ~0.07 units (Ricart et al., 2021); much less than the median 0.16 unit pH impact currently experienced in Tillamook Bay. Expansion of seagrass beyond its present extent in Tillamook Bay may also be limited by present and future constraints on suitable habitat and seagrass physiology. Phytoremediation strategies are therefore likely to be most effective as part of a multi-pronged approach for coastal acidification amelioration in Tillamook Bay. Characterization of the anthropogenic carbon loading rates to Tillamook Bay and other estuaries can be used to inform benchmarks for these suites of carbon removal and amelioration strategies.

The largest water quality impacts in Tillamook Bay as estimated by this study occurred at lower salinities (<13) and were the result of enhanced riverine carbon delivery. These impacts were at times an order of magnitude larger than acidification impacts published for open ocean habitats (Figures 9, 10), and highlight both the increased vulnerability of estuaries to acidification, as well as the within-estuary heterogeneity of acidification impacts. Management strategies for riverine water quality would be most effective in ameliorating acidification in these low salinity and highly impacted areas. The low buffering capacity of waters in these low salinity areas, which presently exacerbates acidification impacts to water quality, also means that carbon removal strategies would result in proportionally large improvements in pH (Cai et al., 2021).

Utilizing existing water quality standards to manage for coastal acidification impacts has been proposed and discussed in the coastal management community for over a decade (Kelly et al., 2011), but the efficacy of this approach is challenged by the fact that conditions in coastal waters often fall within the acceptable range of pH criteria (Weisberg et al., 2016). Our findings in Tillamook Bay underline these difficulties. Despite the estimated acidification impacts in Tillamook Bay, all pH observations during the study period fall within the acceptable range of existing pH water quality standards (6.5–8.5 units) relevant for these waters. It has also been suggested that dissolved oxygen standards in Oregon may be protective for acidification impacts due to their relatively high threshold of 6.5 mg/L ${\text{O}}_{2}$ (Tomasetti and Gobler, 2020). However, our observations show that the lowest ${\text{pH}}_{\text{T}}$ and $\Omega_{\text{arag}}$ conditions in Tillamook Bay, as well as the largest acidification impacts to ${\text{pH}}_{\text{T}}$ and $\Omega_{\text{arag}}$, often occur when dissolved oxygen concentrations are greater than 6.5 mg/L (Supplementary Figure S10).

Tillamook Bay and similar estuarine systems are likely to face increasing OA pressures for decades to come under even the most optimistic climate change mitigation scenarios. Coastal upwelling systems will likely show a delayed response to atmospheric ${\text{CO}}_{2}$ trends due to older water ventilation ages, and therefore water quality benefits from any future global efforts to slow atmospheric ${\text{CO}}_{2}$ growth will not be fully realized in these systems for decades. The dominant influence of OA in Tillamook Bay now and into the future is illustrated by the fact that the additional ${\text{C}}_{\text{ant}}$ delivered to the estuary with only one additional year of OA progression (as of 2017) would exceed the current total anthropogenic carbon loading from the watershed (as observed during this study period). This underlines the importance of management and amelioration strategies capable of addressing impacts from global ${\text{CO}}_{2}$ emissions - whether directly via altered estuarine gas fluxes in systems like the Chesapeake Bay (Da et al., 2021), or via delivery of acidified ocean waters such as seen in Tillamook Bay.

## Data availability statement

The datasets presented in this study can be found in the U.S. EPA’s ScienceHub repository, DOI: 10.23719/1529988.

## Supplementary Material

Supplement1

## Figures and Tables

**TABLE 1:** Watershed characteristics of river systems discharging to the Tillamook Bay, OR.

River	Mean daily discharge (m^3^s^−1^)	Total Watershed Area (km^2^)	Anthropogenic land use					Permitted CAFOs		Human population	
			Total (km^2^)	Upper		Lower		Upper	Lower	Upper	Lower
				km^2^	%	km^2^	%				
**Miami**	6.9	73	6	-	-	6	8%	-	250	-	62
**Kilchis**	13	168	9	4	4%	5	8%	0	875	4	152
**Wilson**	33	489	32	26	6%	5	29%	0	3,091	590	587
**Trask**	28	437	42	17	5%	25	39%	0	11,380	138	6,153
**Tillamook**	6.2	150	27	12	14%	15	25%	825	4,501	707	876

“Upper” anthropogenic land use quantifies the total area of anthropogenic land use above the upstream sampling station for each river, while “Lower” quantifies the area of anthropogenic land use between the lower and upper river sampling stations. Permitted CAFOs indicate the number of permitted cattle in each watershed area.
