# Supplementary material for: Quantifying the combined impacts of anthropogenic CO2 emissions and watershed alteration on estuary acidification at biologically-relevant time scales: a case study from Tillamook Bay, OR, USA
Source: Front Mar Sci. Author manuscript; Available in PMC 2025 Feb 2. (PMC11462966; doi:10.3389/fmars.2024.1293955)
Supplement: Supplement1 [file NIHMS2019219-supplement-Supplement1.docx]

Supplementary Material

Quantifying the combined impacts of anthropogenic CO_2_ emissions and watershed alteration on estuary acidification at biologically-relevant time scales: a case study from Tillamook Bay, OR, USA

**Stephen R. Pacella^*^, Cheryl A. Brown, James E. Kaldy, Rochelle G. Labiosa, Burke Hales, T Chris Mochon Collura, and George G. Waldbusser**

*** Correspondence:** Stephen R. Pacella: [pacella.stephen@epa.gov](mailto:pacella.stephen@epa.gov)

Figure S1. Monthly mean discharges of the Trask River during our 2017-2018 study as compared with the 1994-2014 long-term average. Trask River discharge was ~5% higher during the study period as compared to the long-term average. Data from https://apps.wrd.state.or.us/apps/sw/hydro_report/gage_summary.aspx?station_nbr=14302480&start_date=04/01/1996&end_date=02/17/2014&tolerance=0&fdcCase=usgs&record_status=PUB.

**Extrapolation to zero salinity end-member value for downstream river stations**

During our sampling, some downstream river stations were influenced by tidal advection of estuary waters and therefore did not represent the true river end-member value. In order to estimate the zero salinity river end-member value of DIC and Alk, we used a simple linear regression procedure utilizing the survey-specific coastal ocean end-member sampled at Station M and the observed downriver value. The zero salinity end-member was found as the y-intercept (i.e. when salinity equals zero) of this regression. Below are all regressions utilized for both DIC and Alk.


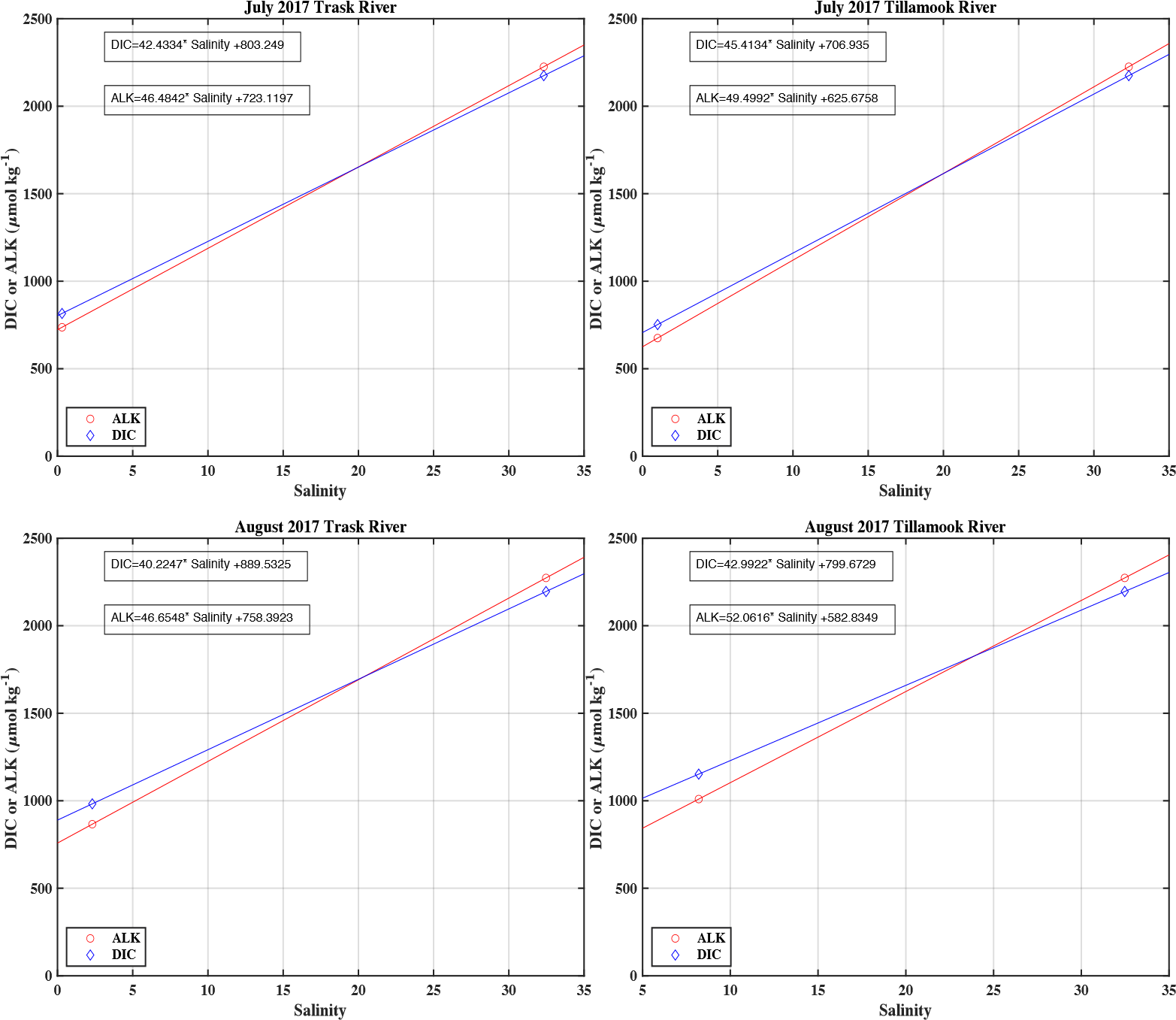

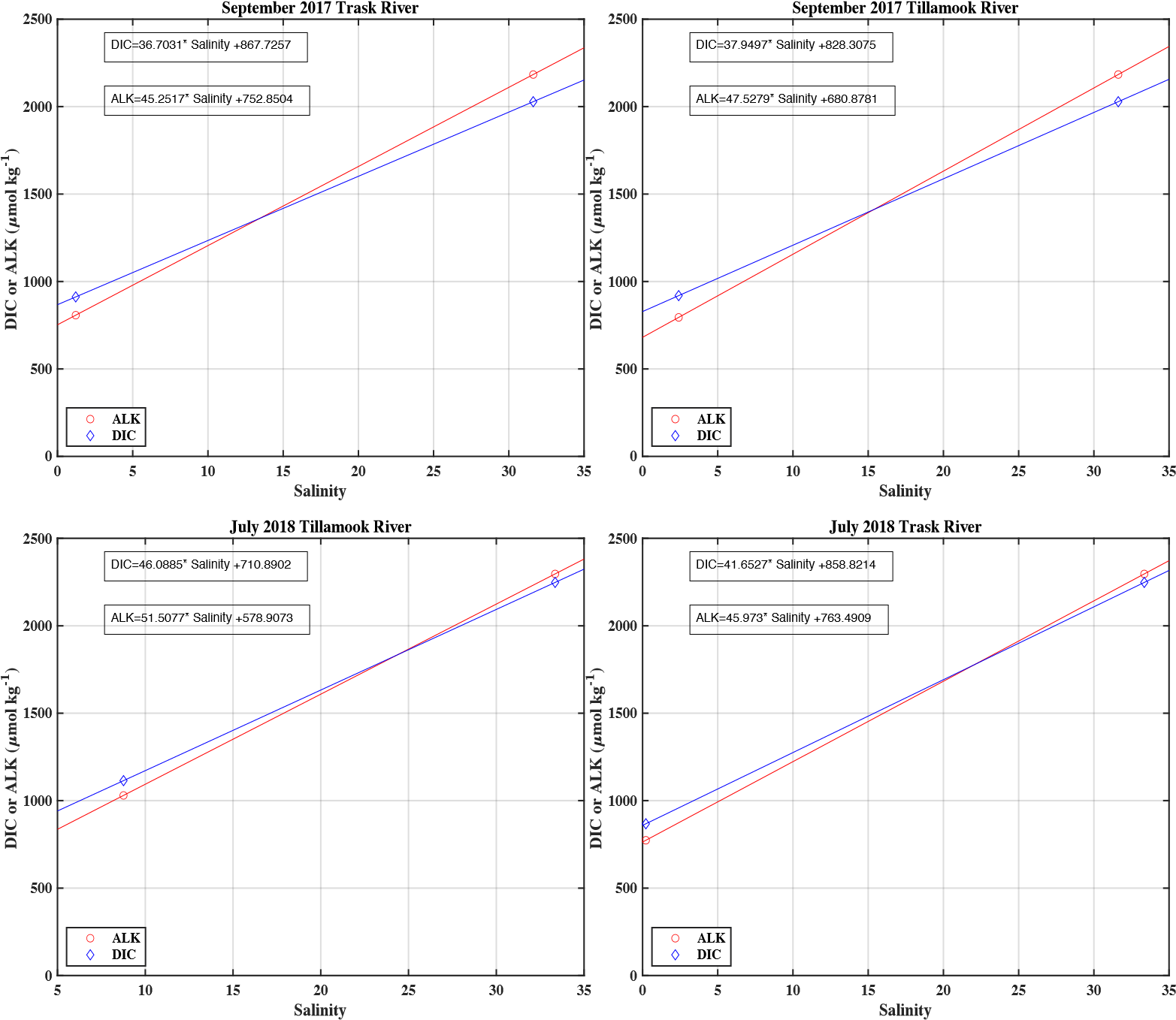


Figure S2. Linear regressions of coastal ocean end-members and downriver station DIC and Alk observations, with observed downriver station salinity > 0.2.


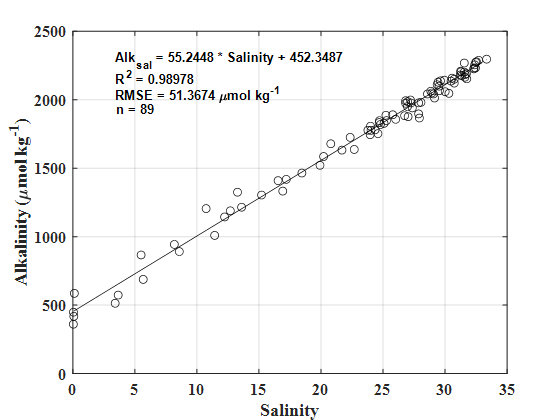


Figure S3. Linear regression of observed salinity and alkalinity from the ten synoptic surveys. Alkalinity was calculated from bottle samples analyzed for both *p*CO_2_ and DIC as in Methods Section 2.3.5
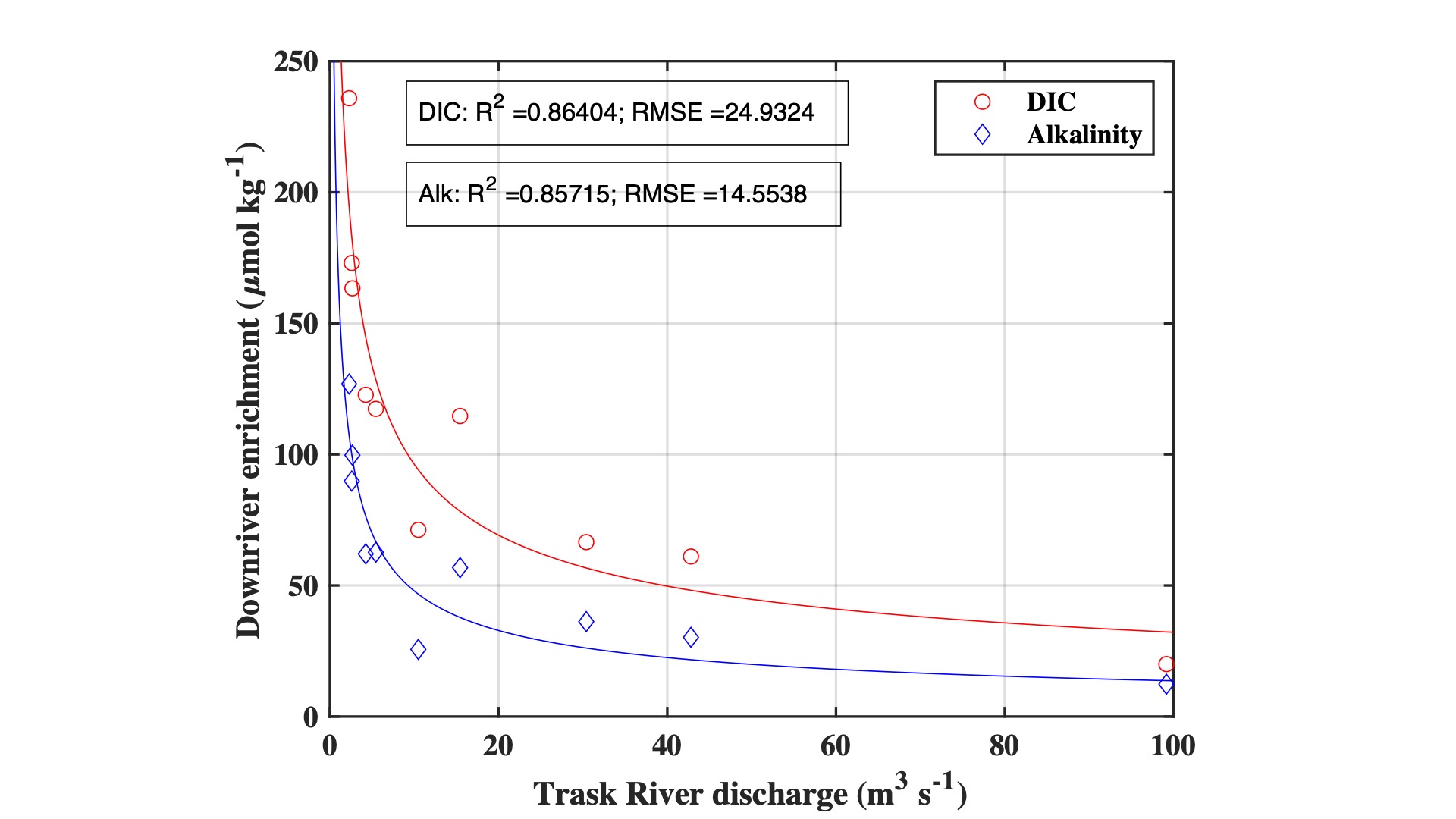


Figure S4. Observed relationships between Trask River discharge on the day of the surveys and flow-weighted average downstream enrichments of river DIC (red) and Alk (blue). Lines shown are power model fits of Trask River discharge with downriver DIC enrichments (red) and downriver Alk enrichments (blue) as explained in Methods 2.4.2.


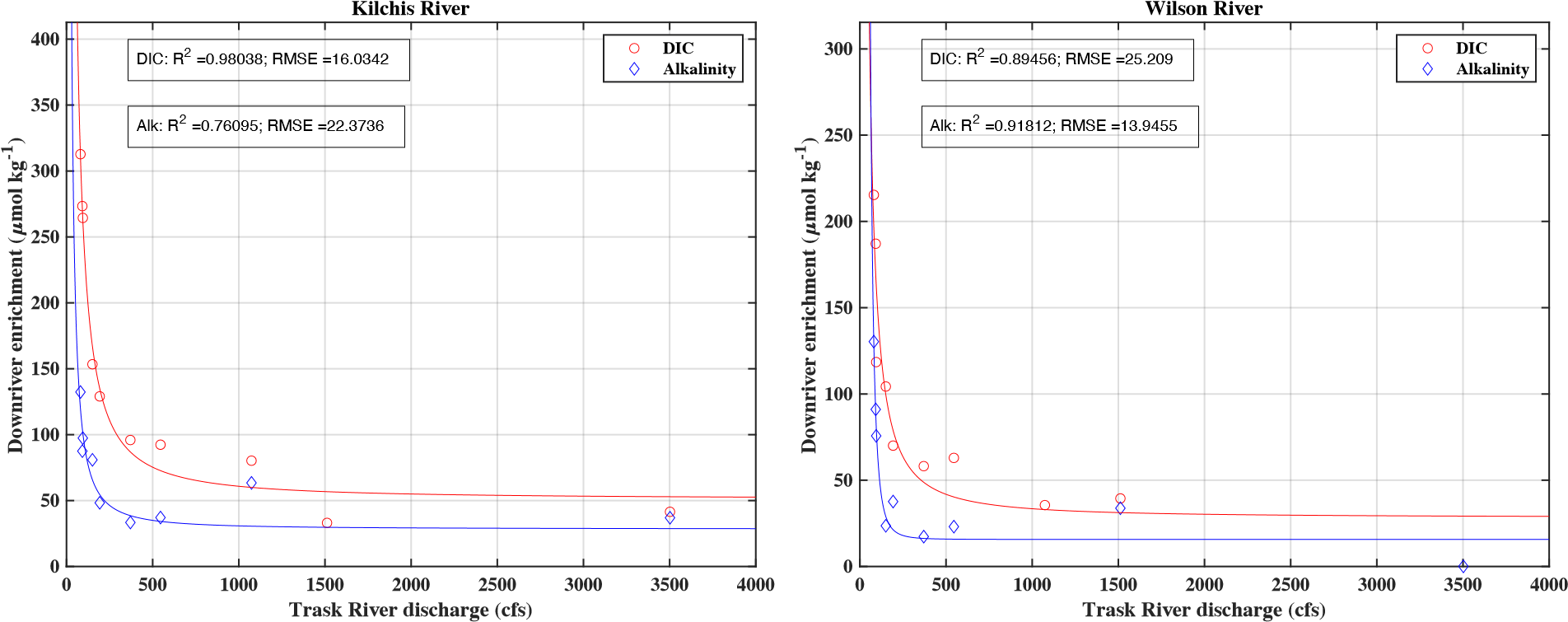

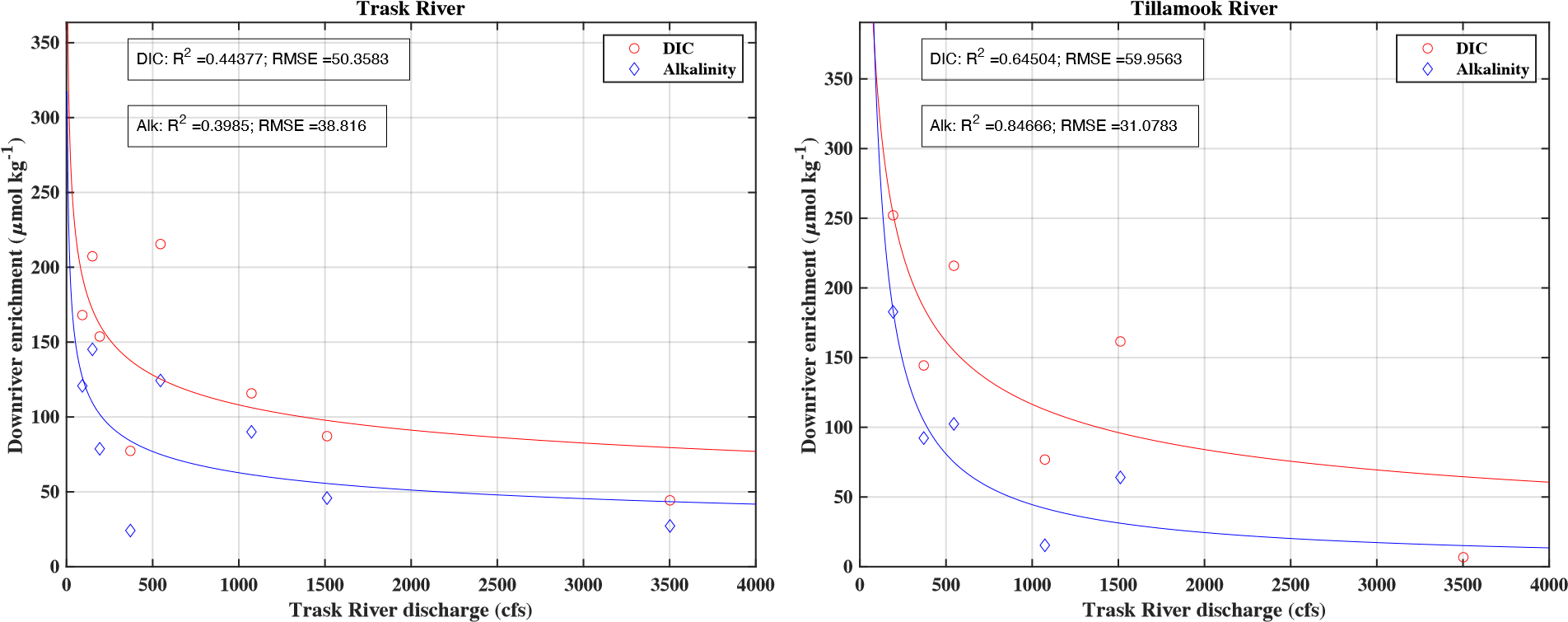


**Figure S5.** River-specific regressions of DIC_enrich_ and Alk_enrich_ with Trask River discharge utilizing only downriver observations with observed salinity <0.2. No extrapolations were used.


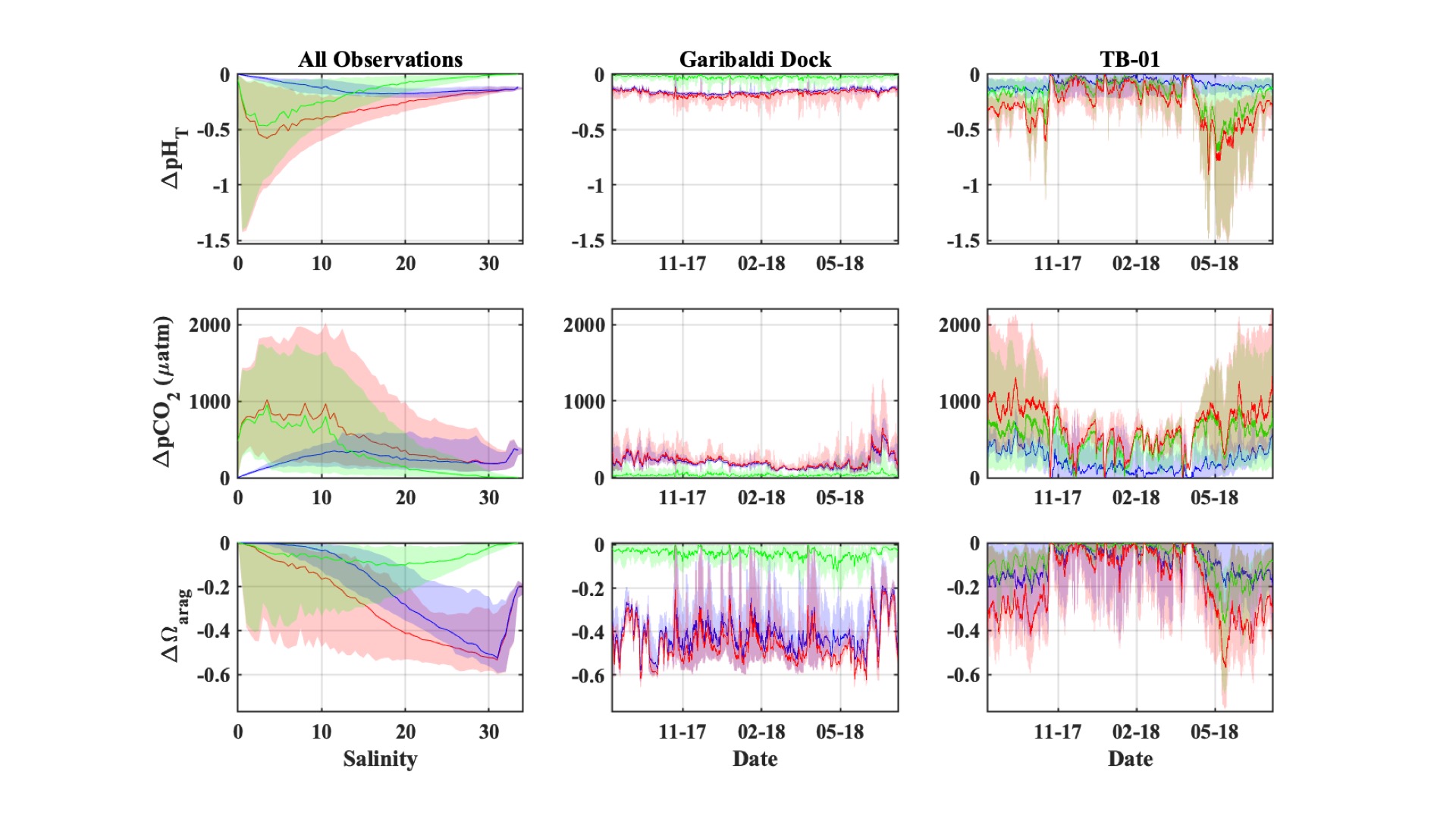


Figure S6. Reproduction of Figure 9 from the manuscript utilizing the river-specific regressions of DIC_enrich_ and Alk_enrich_ shown in Figure S5.


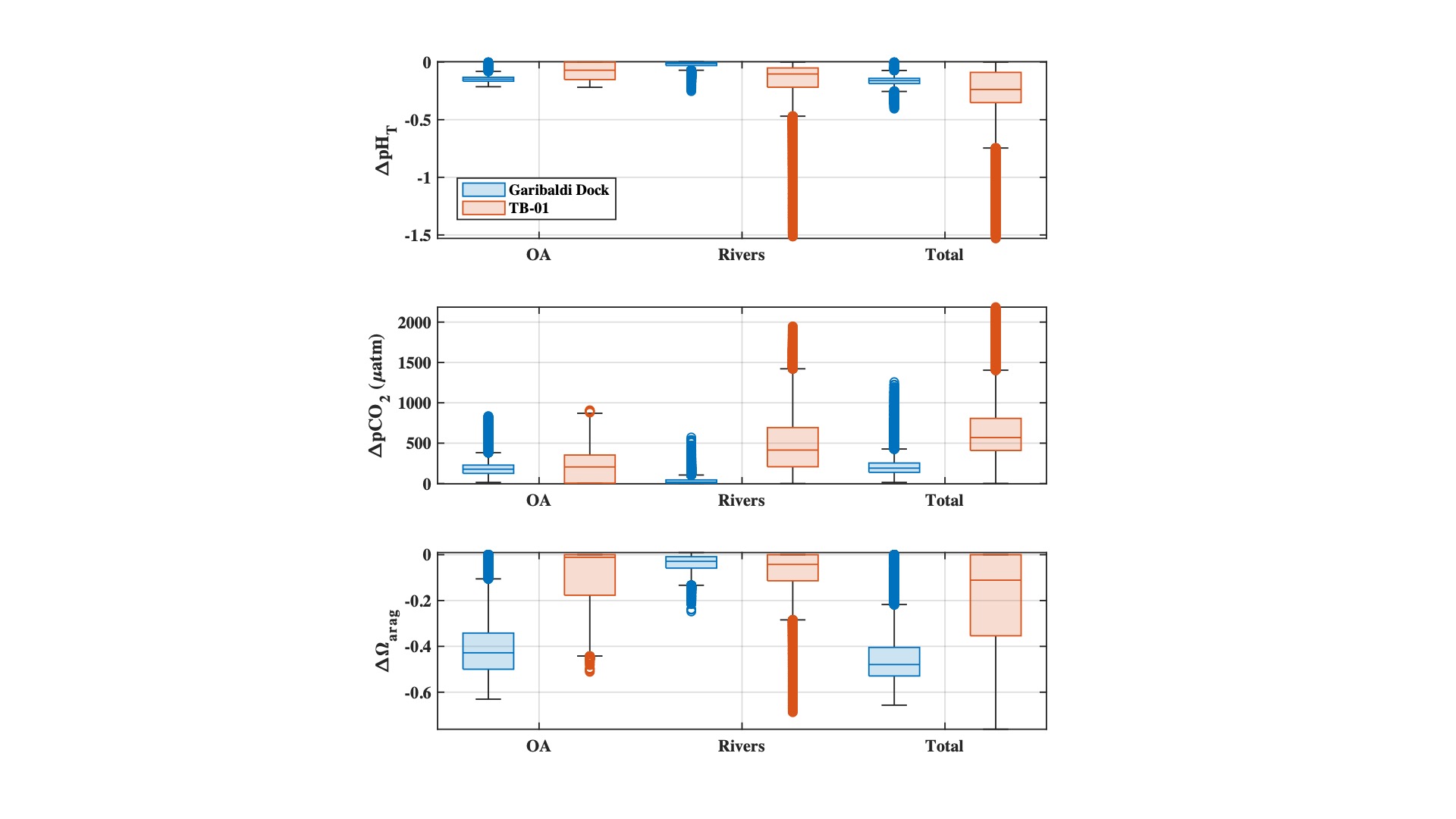


Figure S7. Reproduction of Figure 10 from the manuscript utilizing the river-specific regressions of DIC_enrich_ and Alk_enrich_ shown in Figure S5.


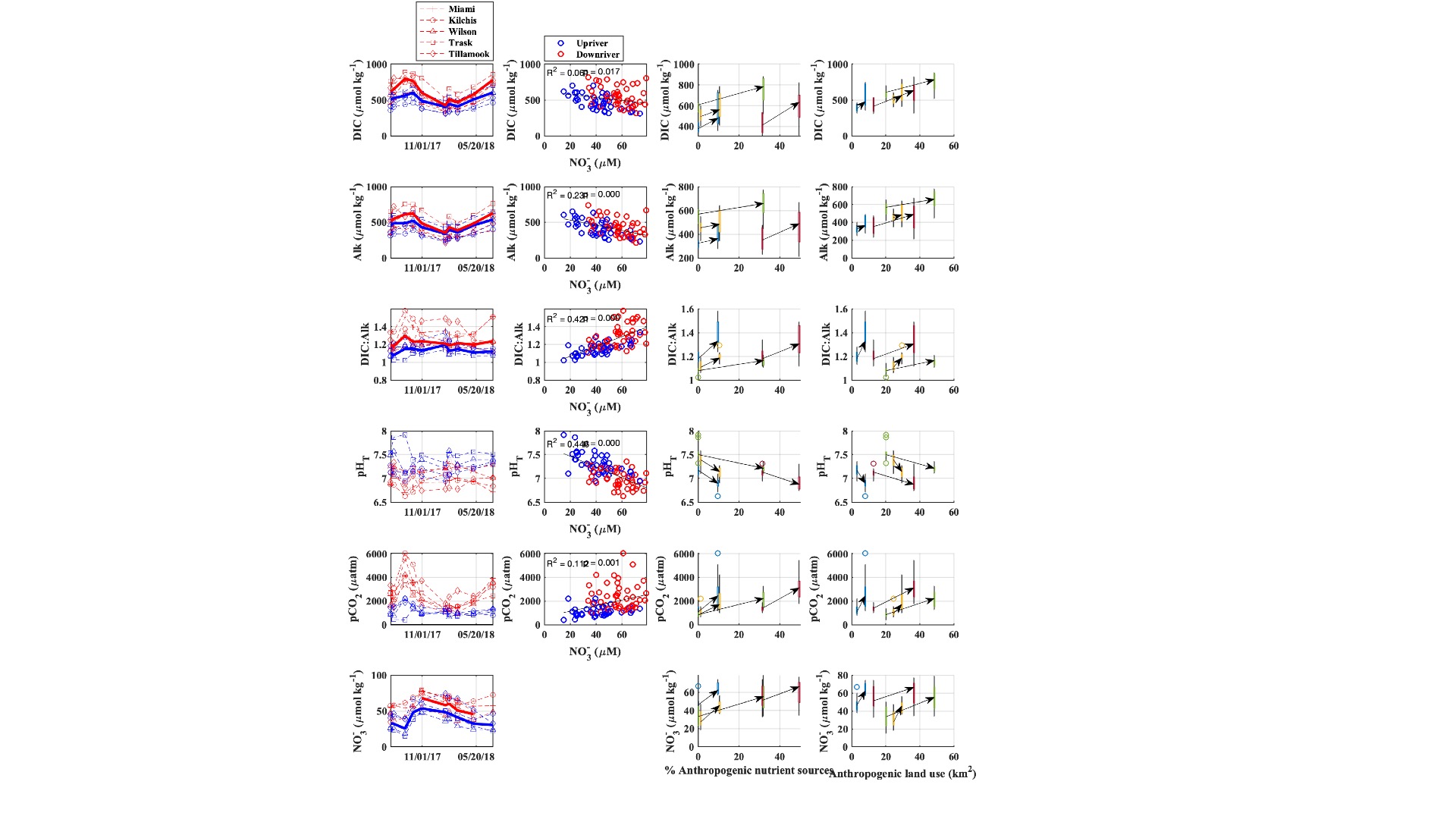


**Figure S8.** Observed carbonate system variables at upstream (blue) and downstream (red) river stations during the synoptic surveys from July 2017 to July 2018, and their relationships with NO3- concentrations, % of anthropogenic nutrient sources from SPARROW model output, and anthropogenic land use area (from MRLC data) in the sub-watershed of each sampling site. Data is color coded by river, and arrows connect upriver to downriver medians for each river.


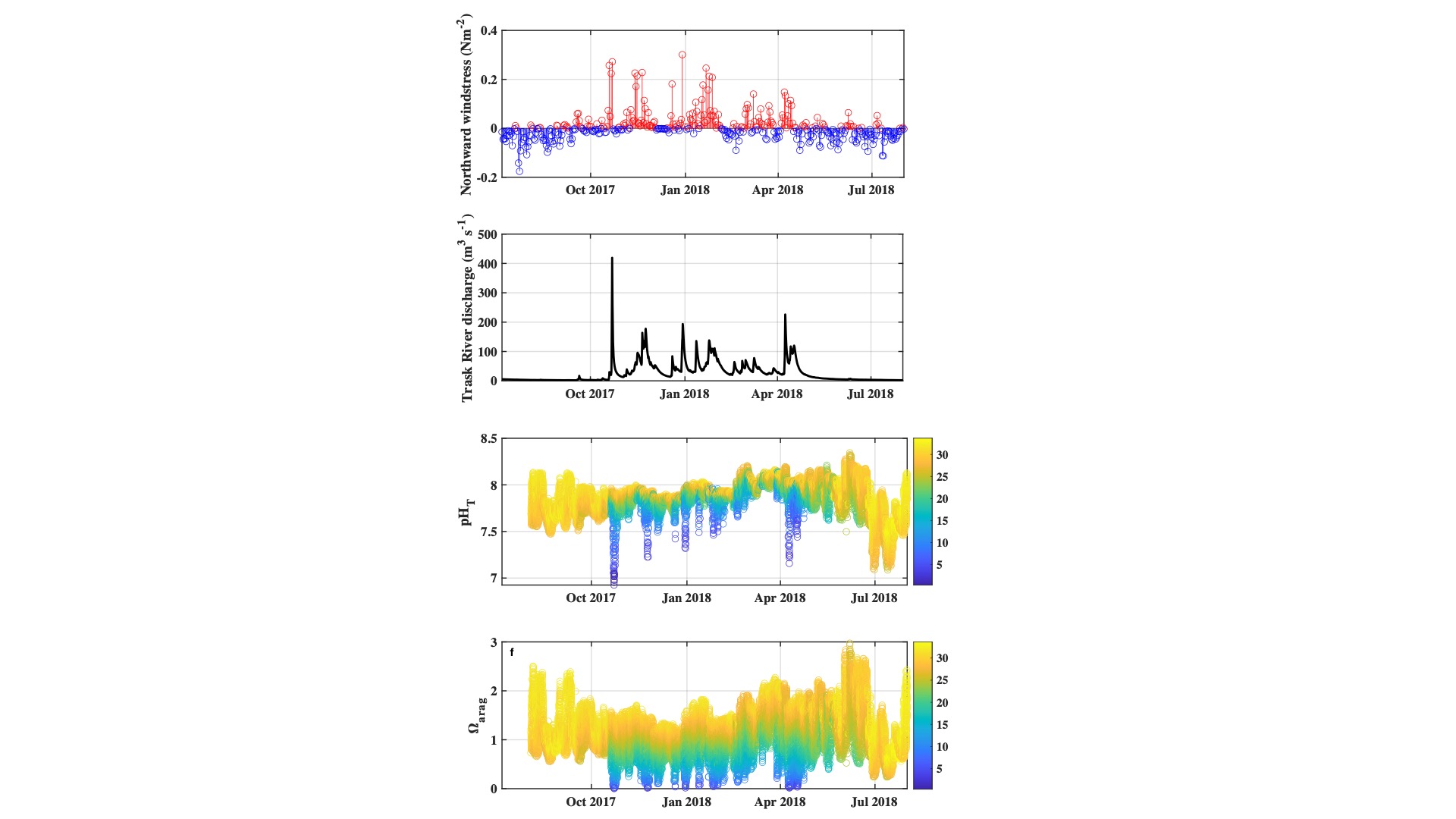


**Figure S9.** Time series of a). Northward wind stress and b). discharge from the Trask River during the study period. Negative (blue) values of northward wind stress indicate upwelling-favorable conditions. Times series from the Garibaldi Dock monitoring station of **c).** observed pH_T_ and d**).** calculated Ω_arag_. Salinity for each observation indicated by shading in legends.


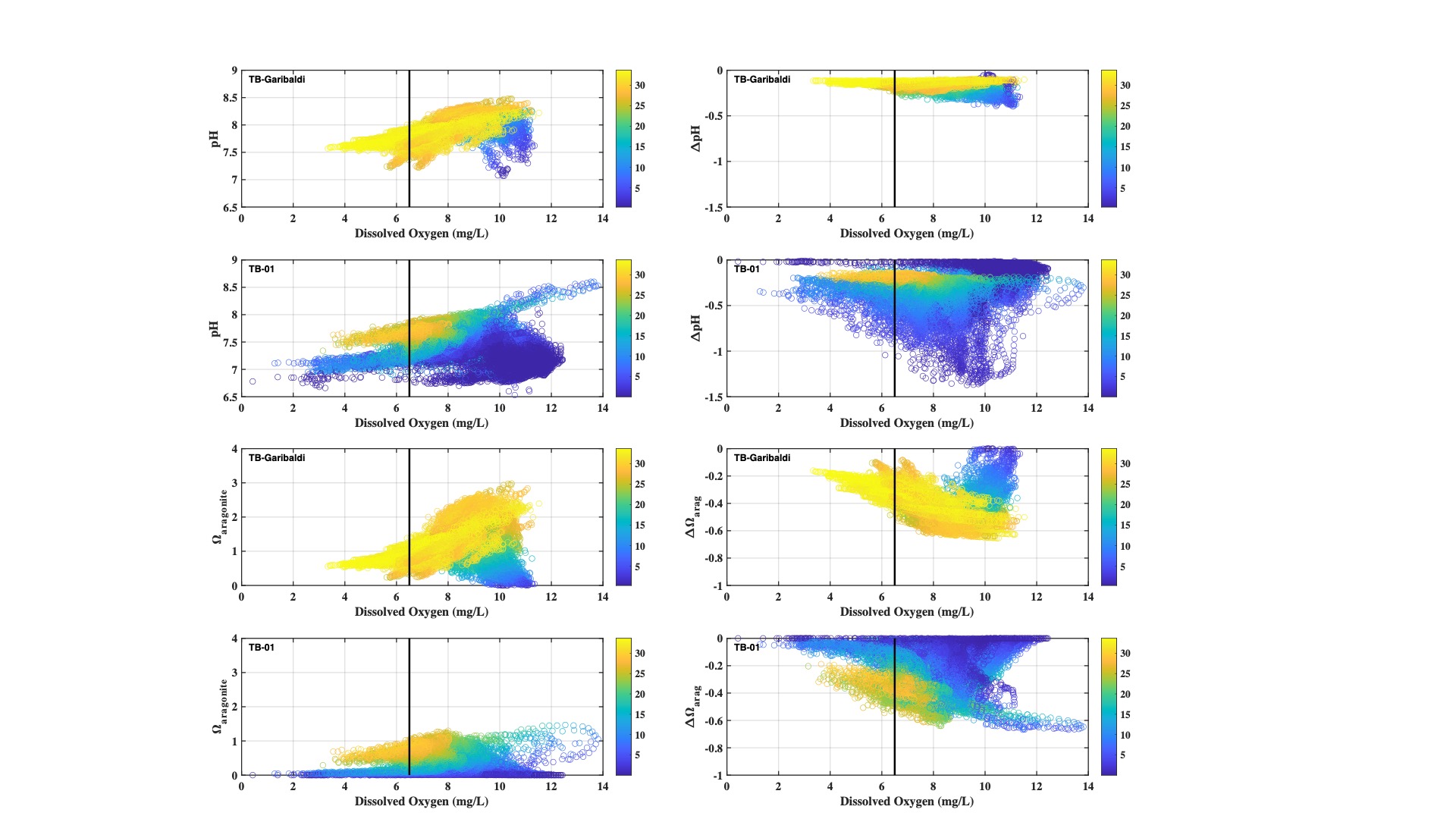


Figure S10. Relationships between dissolved oxygen and observed pH_T_ and Ω_arag_, and acidification impacts to pH_T_ and Ω_arag_, in Tillamook Bay, OR. Color coding corresponds to salinity, and dissolved oxygen standard of 6.5 mg/L is indicated on each plot by black vertical line

**Table S1:** Observed river chemistry from synoptic sampling surveys in 2017 and 2018. Salinity observations with an asterisk (*) indicate a sample that we used to extrapolate back to a zero salinity downriver end-member for DIC and Alk.

|  | **Sampling date** | **Miami River** | **Kilchis River** | | **Wilson River** | | **Trask River** | | **Tillamook River** | | **Flow-weighted Average** | |
| --- | --- | --- | --- | --- | --- | --- | --- | --- | --- | --- | --- | --- |
|  |  |  | Upriver | Downriver | Upriver | Downriver | Upriver | Downriver | Upriver | Downriver | Upriver | Downriver |
| **Salinity** | 7/7/17 | 0.03 | 0.02 | 0.03 | 0.03 | 0.03 | 0.04 | 0.04 | 0.03 | 0.19 | NaN | NaN |
|  | 7/18/17 | 0.03 | 0.02 | 0.03 | 0.03 | 0.04 | 0.04 | 0.3* | 0.03 | 1.0* | NaN | NaN |
|  | 8/9/17 | 0.03 | 0.03 | 0.03 | 0.03 | 0.04 | 0.04 | 2.3* | 0.03 | 8.2* | NaN | NaN |
|  | 9/28/17 | 0.03 | 0.03 | 0.03 | 0.04 | 0.04 | 0.04 | 1.2* | 0.04 | 2.4* | NaN | NaN |
|  | 10/30/17 | 0.03 | 0.02 | 0.03 | 0.03 | 0.03 | 0.04 | 0.08 | 0.03 | 0.12 | NaN | NaN |
|  | 1/25/18 | 0.02 | 0.02 | 0.02 | 0.02 | 0.02 | 0.03 | 0.03 | 0.02 | 0.03 | NaN | NaN |
|  | 2/9/18 | 0.03 | 0.02 | 0.03 | 0.03 | 0.03 | 0.03 | 0.04 | 0.03 | 0.03 | NaN | NaN |
|  | 3/12/18 | 0.03 | 0.02 | 0.02 | 0.02 | 0.03 | 0.03 | 0.03 | 0.03 | 0.04 | NaN | NaN |
|  | 5/10/18 | 0.03 | 0.02 | 0.03 | 0.03 | 0.03 | 0.03 | 0.04 | 0.03 | 0.05 | NaN | NaN |
|  | 7/20/18 | 0.03 | 0.06 | 0.04 | 0.04 | 0.04 | 0.04 | 0.22* | 0.03 | 8.76* | NaN | NaN |
| **Alk (μeq kg-1)** | 7/7/17 | 326 | 316 | 364 | 466 | 504 | 570 | 649 | 352 | 535 | 455 | 518 |
|  | 7/18/17 | 435 | 336 | 417 | 493 | 517 | 592 | 737 | 382 | 618 | 487 | 549 |
|  | 8/9/17 | 395 | 342 | 474 | 470 | 600 | 606 | 756 | 453 | 584 | 487 | 614 |
|  | 9/28/17 | 441 | 390 | 487 | 531 | 606 | 633 | 742 | 455 | 670 | 524 | 623 |
|  | 10/30/17 | 329 | 323 | 361 | 439 | 462 | 543 | 668 | 305 | 408 | 431 | 488 |
|  | 1/25/18 | 250 | 252 | 289 | 347 | 347 | 419 | 446 | 231 | 212 | 340 | 352 |
|  | 2/9/18 | 294 | 280 | 344 | 424 | 419 | 492 | 582 | 271 | 287 | 394 | 431 |
|  | 3/12/18 | 293 | 288 | 278 | 348 | 382 | 449 | 495 | 271 | 335 | 358 | 388 |
|  | 5/10/18 | 343 | 323 | 357 | 443 | 460 | 567 | 591 | 349 | 442 | 458 | 484 |
|  | 7/20/18 | 381 | 398 | 486 | 548 | 639 | 653 | NaN | 476 | 573 | 539 | NaN |
| **DIC (μmol kg^-1^)** | 7/7/17 | 438 | 357 | 486 | 502 | 572 | 609 | 763 | 418 | 670 | 502 | 620 |
|  | 7/18/17 | 505 | 407 | 560 | 523 | 628 | 609 | 816 | 446 | 691 | 526 | 648 |
|  | 8/9/17 | 596 | 437 | 750 | 560 | 775 | 620 | 883 | 533 | 792 | 561 | 797 |
|  | 9/28/17 | 615 | 461 | 726 | 600 | 719 | 698 | 863 | 532 | 823 | 603 | 766 |
|  | 10/30/17 | 439 | 380 | 472 | 488 | 551 | 591 | 806 | 381 | 597 | 488 | 603 |
|  | 1/25/18 | 339 | 316 | 358 | 411 | 410 | 478 | 523 | 309 | 315 | 405 | 426 |
|  | 2/9/18 | 387 | 345 | 426 | 459 | 495 | 538 | 654 | 338 | 415 | 445 | 511 |
|  | 3/12/18 | 377 | 335 | 368 | 403 | 442 | 493 | 580 | 328 | 489 | 410 | 471 |
|  | 5/10/18 | 450 | 374 | 470 | 489 | 547 | 607 | 684 | 407 | 551 | 508 | 578 |
|  | 7/20/18 | 576 | 460 | 733 | 600 | 787 | 700 | NaN | 532 | 700 | 604 | NaN |
| **pH_T_** | 7/7/17 | 6.86 | 7.28 | 6.87 | 7.49 | 7.24 | 7.54 | 7.13 | 7.12 | 6.93 |  |  |
|  | 7/18/17 | 7.21 | 7.08 | 6.86 | 7.57 | 7.04 | 7.86 | 7.29 | 7.15 | 7.30 |  |  |
|  | 8/9/17 | 6.70 | 6.94 | 6.63 | 7.10 | 6.90 | 7.92 | 7.15 | 7.13 | 6.83 |  |  |
|  | 9/28/17 | 6.82 | 7.14 | 6.71 | 7.28 | 7.12 | 7.39 | 7.19 | 7.16 | 7.04 |  |  |
|  | 10/30/17 | 6.91 | 7.20 | 6.95 | 7.39 | 7.15 | 7.49 | 7.11 | 7.05 | 6.74 |  |  |
|  | 1/25/18 | 6.92 | 7.07 | 7.09 | 7.20 | 7.22 | 7.32 | 7.24 | 6.94 | 6.78 |  |  |
|  | 2/9/18 | 6.96 | 7.10 | 7.08 | 7.58 | 7.20 | 7.48 | 7.36 | 7.06 | 6.80 |  |  |
|  | 3/12/18 | 7.01 | 7.27 | 6.96 | 7.28 | 7.27 | 7.48 | 7.22 | 7.14 | 6.77 |  |  |
|  | 5/10/18 | 6.94 | 7.24 | 6.93 | 7.40 | 7.14 | 7.56 | 7.21 | 7.20 | 6.99 |  |  |
|  | 7/20/18 | 6.71 | 7.36 | 6.84 | 7.40 | 7.00 | 7.51 | NaN | 7.31 | 7.03 |  |  |
| ***p*CO_2 (μatm)_** | 7/7/17 | 2434 | 933 | 2672 | 843 | 1648 | 894 | 2718 | 1453 | 3326 |  |  |
|  | 7/18/17 | 1396 | 1508 | 3100 | 756 | 2621 | 446 | 2012 | 1506 | 1910 |  |  |
|  | 8/9/17 | 4192 | 2207 | 6061 | 2219 | 4193 | 414 | 3271 | 1883 | 5429 |  |  |
|  | 9/28/17 | 3543 | 1509 | 5081 | 1523 | 2471 | 1370 | 2858 | 1728 | 3683 |  |  |
|  | 10/30/17 | 2071 | 1051 | 2098 | 917 | 1666 | 882 | 2657 | 1430 | 3716 |  |  |
|  | 1/25/18 | 1529 | 1098 | 1181 | 1092 | 1063 | 993 | 1285 | 1351 | 1780 |  |  |
|  | 2/9/18 | 1647 | 1145 | 1456 | 635 | 1323 | 814 | 1292 | 1186 | 2326 |  |  |
|  | 3/12/18 | 1441 | 785 | 1536 | 919 | 1019 | 737 | 1487 | 1002 | 2888 |  |  |
|  | 5/10/18 | 2025 | 981 | 2206 | 941 | 1757 | 814 | 1924 | 1155 | 2428 |  |  |
|  | 7/20/18 | 3914 | 798 | 3195 | 1278 | 3555 | 1100 | NaN | 1304 | 3386 |  |  |
| **NO_3 (μM)_** | 7/7/17 | 55 | 41 | 57 | 26 | 48 | 25 | 43 | 45 | 35 |  |  |
|  | 7/18/17 | 58 | 42 | 58 | 25 | 37 | 24 | 34 | 46 | NaN |  |  |
|  | 8/9/17 | 54 | 39 | 61 | 19 | 40 | 15 | NaN | 39 | NaN |  |  |
|  | 9/28/17 | 57 | 67 | 69 | 45 | 56 | 38 | NaN | 51 | NaN |  |  |
|  | 10/30/17 | 78 | 60 | 74 | 47 | 54 | 49 | 79 | 66 | 77 |  |  |
|  | 1/25/18 | 68 | 50 | 62 | 36 | 44 | 50 | 68 | 74 | 71 |  |  |
|  | 2/9/18 | 68 | 47 | 71 | 39 | 49 | 44 | 64 | 68 | 69 |  |  |
|  | 3/12/18 | 65 | 47 | 59 | 29 | 37 | 39 | 55 | 67 | 64 |  |  |
|  | 5/10/18 | 57 | 38 | 64 | 24 | 38 | 29 | 44 | 51 | 49 |  |  |
|  | 7/20/18 | 57 | 46 | 72 | 24 | 49 | 22 | NaN | 33 | NaN |  |  |

**Table S2:** Observed estuarine chemistry from synoptic sampling surveys in 2017 and 2018.

|  |  | **Station ID** | | | | | | | | |
| --- | --- | --- | --- | --- | --- | --- | --- | --- | --- | --- |
|  | **Sampling Date** | **1** | **2** | **3** | **4** | **5** | **6** | **7** | **G** | **M** |
| **Alk (μeq kg-1)** | 7/7/17 | 866 | 1,828 | 2,042 | 2,176 | 2,232 | 2,042 | 1,586 | 1,941 | 2,233 |
|  | 7/18/17 | NaN | 1,753 | 1,897 | 2,013 | 2,153 | 1,867 | 1,632 | 1,876 | 2,225 |
|  | 8/9/17 | 1,819 | 2,110 | 2,138 | 2,209 | 2,278 | 2,158 | 1,987 | 2,204 | 2,273 |
|  | 9/28/17 | 1,325 | 1,982 | 2,063 | 2,059 | 2,269 | 2,129 | 1,977 | 2,181 | 2,184 |
|  | 10/30/17 | 943 | 1,847 | 1,637 | 1,778 | 2,047 | 1,807 | 1,522 | 1,977 | 2,136 |
|  | 1/25/18 | 360 | 688 | 1,333 | 891 | 1,009 | 573 | 513 | 1,215 | 2,122 |
|  | 2/9/18 | 448 | 1,777 | 1,679 | 1,857 | 2,099 | 1,950 | 1,780 | 1,884 | 2,165 |
|  | 3/12/18 | 418 | 1,144 | 1,189 | 1,304 | 1,998 | 1,409 | 1,419 | 1,887 | 2,144 |
|  | 5/10/18 | 586 | 1,745 | 1,848 | 1,891 | 1,974 | 1,833 | 1,465 | 1,725 | 1,993 |
|  | 7/20/18 | NaN | 2,067 | 2,202 | 2,255 | 2,289 | 2,191 | 2,050 | 2,150 | 2,297 |
| **DIC (μmol kg^-1^)** | 7/7/17 | 921 | 1,726 | 1,934 | 2,047 | 2,086 | 1,902 | 1,501 | 1,826 | 2,133 |
|  | 7/18/17 | NaN | 1,675 | 1,814 | 1,915 | 2,079 | 1,777 | 1,564 | 1,803 | 2,175 |
|  | 8/9/17 | 1,780 | 2,056 | 2,094 | 2,143 | 2,216 | 2,099 | 1,929 | 2,145 | 2,196 |
|  | 9/28/17 | 1,361 | 1,885 | 1,936 | 1,938 | 2,079 | 1,991 | 1,871 | 2,018 | 2,028 |
|  | 10/30/17 | 1,002 | 1,761 | 1,592 | 1,710 | 1,908 | 1,718 | 1,483 | 1,870 | 1,978 |
|  | 1/25/18 | 462 | 734 | 1,311 | 878 | 1,024 | 603 | 543 | 1,188 | 1,971 |
|  | 2/9/18 | 573 | 1,690 | 1,608 | 1,755 | 1,953 | 1,814 | 1,688 | 1,779 | 1,993 |
|  | 3/12/18 | 528 | 1,114 | 1,163 | 1,256 | 1,858 | 1,342 | 1,353 | 1,757 | 1,975 |
|  | 5/10/18 | 676 | 1,650 | 1,738 | 1,750 | 1,779 | 1,703 | 1,409 | 1,621 | 1,793 |
|  | 7/20/18 | NaN | 2,053 | 2,181 | 2,241 | 2,254 | 2,120 | 1,994 | 2,085 | 2,248 |
| **pH_T_** | 7/7/17 | 7.27 | 7.91 | 7.88 | 7.94 | 7.99 | 7.99 | 7.93 | 7.93 | 7.87 |
|  | 7/18/17 | NaN | 7.82 | 7.81 | 7.85 | 7.79 | 7.83 | 7.81 | 7.77 | 7.72 |
|  | 8/9/17 | 7.63 | 7.67 | 7.64 | 7.73 | 7.74 | 7.69 | 7.70 | 7.70 | 7.79 |
|  | 9/28/17 | 7.37 | 7.84 | 7.92 | 7.91 | 8.06 | 7.94 | 7.87 | 8.00 | 7.99 |
|  | 10/30/17 | 7.23 | 7.91 | 7.79 | 7.87 | 7.99 | 7.94 | 7.80 | 7.94 | 8.03 |
|  | 1/25/18 | 7.01 | 7.36 | 7.81 | 7.96 | 7.63 | 7.50 | 7.47 | 7.92 | 8.04 |
|  | 2/9/18 | 7.00 | 7.97 | 7.96 | 7.98 | 8.04 | 8.07 | 7.98 | 7.98 | 8.07 |
|  | 3/12/18 | 7.01 | 7.96 | 7.92 | 7.99 | 8.11 | 8.02 | 8.01 | 8.07 | 8.09 |
|  | 5/10/18 | 7.20 | 7.94 | 7.96 | 8.04 | 8.18 | 8.03 | 7.88 | 7.99 | 8.19 |
|  | 7/20/18 | NaN | 7.76 | 7.80 | 7.77 | 7.66 | 7.73 | 7.68 | 7.71 | 7.70 |
| ***p*CO_2 (μatm)_** | 7/7/17 | 1543 | 485 | 573 | 512 | 449 | 431 | 430 | 480 | 612 |
|  | 7/18/17 | NaN | 604 | 644 | 605 | 723 | 602 | 588 | 694 | 888 |
|  | 8/9/17 | 1000 | 995 | 1083 | 889 | 859 | 946 | 882 | 936 | 756 |
|  | 9/28/17 | 1509 | 612 | 522 | 530 | 387 | 507 | 568 | 434 | 449 |
|  | 10/30/17 | 1408 | 482 | 595 | 529 | 424 | 447 | 554 | 478 | 401 |
|  | 1/25/18 | 1740 | 898 | 495 | 255 | 603 | 584 | 568 | 354 | 393 |
|  | 2/9/18 | 2248 | 407 | 401 | 400 | 383 | 339 | 392 | 412 | 359 |
|  | 3/12/18 | 2044 | 309 | 354 | 308 | 313 | 296 | 308 | 323 | 336 |
|  | 5/10/18 | 1976 | 438 | 433 | 352 | 256 | 356 | 453 | 382 | 251 |
|  | 7/20/18 | NaN | 943 | 888 | 919 | 1053 | 872 | 943 | 899 | 952 |
| **Ω_arag_** | 7/7/17 | 0.1 | 1.4 | 1.4 | 1.6 | 1.7 | 1.7 | 1.1 | 1.5 | 1.3 |
|  | 7/18/17 | NaN | 1.1 | 1.1 | 1.3 | 1.1 | 1.2 | 1.1 | 1.1 | 0.9 |
|  | 8/9/17 | 0.9 | 1.0 | 0.9 | 1.1 | 1.0 | 1.0 | 1.0 | 1.0 | 1.1 |
|  | 9/28/17 | 0.3 | 1.3 | 1.6 | 1.5 | 2.2 | 1.7 | 1.4 | 1.9 | 1.8 |
|  | 10/30/17 | 0.1 | 1.2 | 0.8 | 1.0 | 1.6 | 1.2 | 0.7 | 1.4 | 1.8 |
|  | 1/25/18 | 0.0 | 0.1 | 0.6 | 0.4 | 0.2 | 0.1 | 0.1 | 0.6 | 1.7 |
|  | 2/9/18 | 0.0 | 1.2 | 1.1 | 1.3 | 1.7 | 1.6 | 1.2 | 1.3 | 1.9 |
|  | 3/12/18 | 0.0 | 0.6 | 0.6 | 0.8 | 1.7 | 1.0 | 1.0 | 1.6 | 1.9 |
|  | 5/10/18 | 0.1 | 1.3 | 1.4 | 1.7 | 2.3 | 1.6 | 0.9 | 1.4 | 2.3 |
|  | 7/20/18 | NaN | 0.9 | 1.0 | 0.9 | 0.8 | 1.1 | 1.0 | 1.1 | 0.9 |
| **Salinity** | 7/7/17 | 5.5 | 25.1 | 29.1 | 31.4 | 32.3 | 28.6 | 20.2 | 27.4 | 32.5 |
|  | 7/18/17 | NaN | 24.6 | 27.9 | 29.2 | 31.8 | 27.9 | 21.7 | 27.0 | 32.3 |
|  | 8/9/17 | 24.8 | 29.4 | 29.6 | 31.3 | 32.5 | 30.6 | 27.0 | 31.3 | 32.5 |
|  | 9/28/17 | 13.3 | 28.1 | 28.9 | 30.0 | 31.6 | 29.4 | 27.9 | 31.2 | 31.6 |
|  | 10/30/17 | 8.2 | 24.7 | 22.7 | 23.8 | 30.3 | 24.0 | 19.9 | 27.3 | 30.5 |
|  | 1/25/18 | 0.0 | 5.7 | 16.9 | 8.6 | 11.4 | 3.7 | 3.4 | 13.6 | 30.7 |
|  | 2/9/18 | 0.1 | 24.0 | 20.8 | 26.0 | 29.5 | 27.0 | 24.4 | 26.7 | 31.6 |
|  | 3/12/18 | 0.1 | 12.2 | 12.7 | 15.2 | 27.2 | 16.5 | 17.2 | 25.2 | 29.9 |
|  | 5/10/18 | 0.1 | 24.0 | 25.3 | 25.8 | 26.8 | 24.7 | 18.5 | 22.4 | 26.8 |
|  | 7/20/18 | 10.7 | 29.6 | 31.6 | 32.4 | 32.7 | 31.7 | 28.9 | 30.7 | 33.4 |
